# Supplementary material for: Schwann cell plasticity regulates neuroblastic tumor cell differentiation via epidermal growth factor-like protein 8
Source: Nat Commun. 2021 Mar 12;12:1624. doi: 10.1038/s41467-021-21859-0 (PMC7954855; doi:10.1038/s41467-021-21859-0)
Supplement: Supplementary file 1 — Supplementary Information [file 41467_2021_21859_MOESM1_ESM.pdf]

SUPPLEMENTARY INFORMATION

SUPPLEMENTARY TABLES

						Transcriptomics, RNA-seq 50bp		
				Proteomics, hr-MS				
	CLB-Ma *	IMR5 *	SH-SY5Y *	STA-NB-10 *,#	STA-NB-2#	STA-NB-7#	STA-NB-6 *,#	STA-NB-15#
INSS stage	4	NA	4	3	4	3	3	4
patient outcome	NA	NA	DOD	DOD	CR	DOD	CR	DOD
MNA	yes dmin	yes HSR	no	yes dmin	no	yes dmin	no	yes dmin
ploidy	diploid	NA	diploid	di-/tetra-ploid	diploid	di-/tetra-ploid	aneuploid	diploid
17q gain	yes	NA	yes	yes	yes	yes	Yes	yes
1p loss	yes	yes	no	yes	yes	yes	yes	yes
11q loss	no	yes	no	no	yes	no	wcUPD	no
reference	1	2, 3	4, 5	6, 7	6, 7	6, 7	6, 7	6, 7

Supplementary table 1 | NBT cell lines characteristics

INSS (International Neuroblastoma Staging System), NA (not available), MNA (*MYCN* amplification), HSR (homogeneously staining regions), WT (wild type), DOD (death of disease), CR (complete remission), dmin (double minutes), wcUPD (whole chromosome uniparental disomy), *cell lines/cultures were used for co-culture with primary repair-related SCs; #primary short-term NB cultures.

	Transcriptomics, RNA-sequencing																	
															Proteomics, hrMS			
	NB-TU02	NB-TU 05	NB-TU 06	NB-TU 44	NB-TU 47	NB-TU 48	NB-TU 49	NB-TU 50	NB-TU 52	NB-TU 53	NB-TU 54	NB-TU 55	NB-TU 57	NB-TU 62	NB-TU 63	NB-TU 80	NB-TU 81	NB-TU 148
dia-gnostic	y	y	y	y	y	y	y	y	y	y	y	y	y	y	y	y	y	y
INSS stage	4	4	4	4	4	4	4	4	4	4	4	4	4	4	4	4	3	3
DOD	NA	n	y	n	NA	y	n	n	y	y	y	y	n	y	y	n	n	y
MNA	y	y	n	y	y	y	y	n	y	y	y	n	y	n	n	y	y	y
17q gain	NA	y	y	y	NA	y	y	y	n	y	NA	y	y	y	n	y	y	y
1p loss	y	n	n	y	NA	y	y	n	y	y	NA	y	y	n	n	y	y	y
11q loss	NA	UPD	y	n	NA	n	n	y	n	n	NA	y	n	y	y	n	n	n

Supplementary table 2 | NB-TU characteristics

INSS (International Neuroblastoma Staging System), y (yes), n (no), NA (not available), MNA (*MYCN* amplification), DOD (death of disease), UPD (uniparental disomy).

1st antibodies					Application					
					Immunofluorescence		Flow cytometry		Western Blot	
Antigen	Species	Catalog No	Company	Clone name	Dilution	Comment	Dilution	Comment	Dilution	Comment
S100B	rabbit	#Z0311	DAKO	polyclonal	1:200	1 hr, RT, perm				
Ki67	mouse	NCL-Ki67-MM1	Leica Microsystems	MM1	1:50	1 hr, RT, perm				
Sox10	mouse	Sc-365692	Santa Cruz	A-2	1:50	o.n., 4°C, perm				
vimentin	mouse	#M0725	DAKO	V9			1:200	20 min, 4°C, perm		
vimentin	chicken	#AB5733	Merck Millipore	polyclonal	1:200	1 hr, RT, perm				
NF200	mouse	MAB5266	Merck Milipore	N52	1:200	1 hr, RT, perm				
NGFR	rabbit	#8238S	CellSignaling	D4B3	1:300	o.n., 4°C				
S100B-FITC*	rabbit	#Z0311	DAKO	polyclonal			1:50	20 min, 4°C, perm		
GD2-FITC*	humanized chinese hamster	ch14:18	POLYMUN GmbH	ch14:18	1:70	o.n., 4°C				
GD2-A546*	humanized chinese hamster	ch14:18	POLYMUN GmbH	ch14:18	1:70	o.n., 4°C	1:1000	20 min, 4°C		
NF200-A647*	mouse	MAB5266	Merck Milipore	N52			1:400	20 min, 4°C, perm		
EGFL8	rabbit	#PA5-63929	Thermo Scientific	polyclonal	1:100	o.n., 4°C, perm			1:333	o.n., 4°C
CD3	mouse	#C7048	Sigma-Aldrich	UCHT-1	1:50	o.n., 4°C				
HLA-DR-α1	mouse	#M0746	DAKO	TAL-1B5	1:50	o.n., 4°C				
c-JUN	rabbit	#9165	CellSignaling	60A8	1:100	o.n., 4°C perm				
GAPDH	mouse	#32233	Santa Cruz	6C5					1:2000	1h, RT

2nd antibodies					Application					
					Immunofluorescence		Flow cytometry		Western Blot	

Antigen	Species	Catalog No	Company	Clone name	Dilution	Comment	Dilution	Comment	Dilution	Comment
α rb FITC	swine	#F0205	DAKO	polyclonal	1:50	1 hr, RT				
α ms AF594	goat	#A11032	LifeTech.	polyclonal	1:300	1 hr, RT	1:1000	20 min, 4°C		
α ch AF647	goat	#SA5-10073	LifeTech.	polyclonal	1:300	1 hr, RT				
α ms IRdye680LT	goat	P/N 925-68020	LI-COR	polyclonal					1:10000	1h, RT
α rb HRP	goat	#7074	Cell Signaling	polyclonal					1:1000	10 min, RT

Recombinant protein			Application	
Protein	Catalog No	Company	Stock concentration	Final concentration
[illegible]-NGF	450-01	Peprotech	25 mg/mL	20 ng/mL
BDNF	450-02	Peprotech	10 mg/mL	40 ng/mL
CNTF	450-13	Peprotech	12.5 mg/mL	10 ng/ml
PTN	450-15	Peprotech	25 mg/mL	50 ng/ml
GDNF	450-10	Peprotech	10 mg/mL	30 ng/ml
IGFBP6	350-07B	Peprotech	25 mg/mL	100 ng/ml
FGF7	130-093-849	Miltenyi Biotech	25 mg/mL	100 ng/ml
EGFL8	H00080864-P01	Abnova	90 mg/mL	20 – 100 ng/ml

Supplementary table 3│ List of antibodies and recombinant proteins.

*manually labeled, perm = permeabilization necessary, RT = room temperature.

RNA-Seq sample	GEO series	Sample identifier	Description	URL/DOI
STA-NB-6	GSE90711	GSM2445387	Neuroblastoma cell line STA-NB-6 (3) (replacement BAM)	https://www.ncbi.nlm.nih.gov/geo/query/acc.cgi?acc=GSE90711
STA-NB-7	GSE90711	GSM2445389	Neuroblastoma cell line STA-NB-7 (replacement BAM)	https://www.ncbi.nlm.nih.gov/geo/query/acc.cgi?acc=GSE90711
STA-NB-15	GSE90711	GSM2445390	Neuroblastoma cell line STA-NB-15 (replacement BAM)	https://www.ncbi.nlm.nih.gov/geo/query/acc.cgi?acc=GSE90711
NB-TU 02	GSE94035	GSM2467430	Tumor at diagnosis. Patient p02	https://www.ncbi.nlm.nih.gov/geo/query/acc.cgi?acc=GSE94035
NB-TU 05	GSE94035	GSM2467431	Tumor at diagnosis. Patient p05	https://www.ncbi.nlm.nih.gov/geo/query/acc.cgi?acc=GSE94035
NB-TU 06	GSE94035	GSM2467432	Tumor at diagnosis. Patient p06	https://www.ncbi.nlm.nih.gov/geo/query/acc.cgi?acc=GSE94035
NB-TU 44	GSE94035	GSM2467433	Tumor at diagnosis. Patient p44	https://www.ncbi.nlm.nih.gov/geo/query/acc.cgi?acc=GSE94035
NB-TU 47	GSE94035	GSM2467434	Tumor at diagnosis. Patient p47	https://www.ncbi.nlm.nih.gov/geo/query/acc.cgi?acc=GSE94035
NB-TU 48	GSE94035	GSM2467435	Tumor at diagnosis. Patient p48	https://www.ncbi.nlm.nih.gov/geo/query/acc.cgi?acc=GSE94035
NB-TU 49	GSE94035	GSM2467436	Tumor at diagnosis. Patient p49	https://www.ncbi.nlm.nih.gov/geo/query/acc.cgi?acc=GSE94035
NB-TU 50	GSE94035	GSM2467437	Tumor at diagnosis. Patient p50	https://www.ncbi.nlm.nih.gov/geo/query/acc.cgi?acc=GSE94035
NB-TU 52	GSE94035	GSM2467438	Tumor at diagnosis. Patient p52	https://www.ncbi.nlm.nih.gov/geo/query/acc.cgi?acc=GSE94035
NB-TU 53	GSE94035	GSM2467439	Tumor at diagnosis. Patient p53	https://www.ncbi.nlm.nih.gov/geo/query/acc.cgi?acc=GSE94035
NB-TU 54	GSE94035	GSM2467440	Tumor at diagnosis. Patient p54	https://www.ncbi.nlm.nih.gov/geo/query/acc.cgi?acc=GSE94035
NB-TU 55	GSE94035	GSM2467441	Tumor at diagnosis. Patient p55	https://www.ncbi.nlm.nih.gov/geo/query/acc.cgi?acc=GSE94035
NB-TU 57	GSE94035	GSM2467443	Tumor at diagnosis. Patient p57	https://www.ncbi.nlm.nih.gov/geo/query/acc.cgi?acc=GSE94035
NB-TU 62	GSE94035	GSM2467444	Tumor at diagnosis. Patient p62	https://www.ncbi.nlm.nih.gov/geo/query/acc.cgi?acc=GSE94035
NB-TU 63	GSE94035	GSM2467445	Tumor at diagnosis. Patient p63	https://www.ncbi.nlm.nih.gov/geo/query/acc.cgi?acc=GSE94035
SC-GN 1	GSE147635	GSM4437031	Ganglioneuroma 1	https://www.ncbi.nlm.nih.gov/geo/query/acc.cgi?acc=GSE147635
SC-GN 2	GSE147635	GSM4437032	Ganglioneuroma 2	https://www.ncbi.nlm.nih.gov/geo/query/acc.cgi?acc=GSE147635
SC-GN 3	GSE147635	GSM4437033	Ganglioneuroma 3	https://www.ncbi.nlm.nih.gov/geo/query/acc.cgi?acc=GSE147635
SC-GN 4	GSE147635	GSM4437034	Ganglioneuroma 4	https://www.ncbi.nlm.nih.gov/geo/query/acc.cgi?acc=GSE147635
SC-GN 5	GSE147635	GSM4437035	Ganglioneuroma 5	https://www.ncbi.nlm.nih.gov/geo/query/acc.cgi?acc=GSE147635
SC-GN 6	GSE147635	GSM4437036	Ganglioneuroma 6	https://www.ncbi.nlm.nih.gov/geo/query/acc.cgi?acc=GSE147635
SC-IN 1	GSE90711	GSM2445374	Peripheral nerve fascicle injured 1	https://www.ncbi.nlm.nih.gov/geo/query/acc.cgi?acc=GSE90711
SC-IN 2	GSE90711	GSM2445375	Peripheral nerve fascicle injured 2	https://www.ncbi.nlm.nih.gov/geo/query/acc.cgi?acc=GSE90711
SC-IN 3	GSE90711	GSM2445376	Peripheral nerve fascicle injured 3	https://www.ncbi.nlm.nih.gov/geo/query/acc.cgi?acc=GSE90711
SC 1	GSE90711	GSM2445377	Peripheral nerve associated primary Schwann cells 1	https://www.ncbi.nlm.nih.gov/geo/query/acc.cgi?acc=GSE90711
SC 2	GSE90711	GSM2445378	Peripheral nerve associated primary Schwann cells 2	https://www.ncbi.nlm.nih.gov/geo/query/acc.cgi?acc=GSE90711
SC 3	GSE90711	GSM2445379	Peripheral nerve associated primary Schwann cells 3	https://www.ncbi.nlm.nih.gov/geo/query/acc.cgi?acc=GSE90711
SC 4	GSE90711	GSM2445380	Peripheral nerve associated primary Schwann cells 4	https://www.ncbi.nlm.nih.gov/geo/query/acc.cgi?acc=GSE90711
SC 5	GSE90711	GSM2445381	Peripheral nerve associated primary Schwann cells 5	https://www.ncbi.nlm.nih.gov/geo/query/acc.cgi?acc=GSE90711
Dataset Kocak	GSE45547		Kocak neuroblastoma tumor transcriptomics dataset (n=649)	https://www.ncbi.nlm.nih.gov/geo/query/acc.cgi?acc=GSE45547
Dataset NRC	GSE85047		NRC neuroblastoma tumor transcriptomics dataset (n=283)	https://www.ncbi.nlm.nih.gov/geo/query/acc.cgi?acc=GSE85047

Supplementary table 4| Data repository identifier for samples used in this study. RNA-sequencing datasets were uploaded to the gene expression omnibus (GEO) repository (https://www.ncbi.nlm.nih.gov/geo/).

Category	Term	% of genes	p-value
UP_KEYWORDS	Immunity	7,1	7,8E-22
UP_KEYWORDS	Glycoprotein	30,8	1,1E-19
GOTERM_BP_DIRECT	inflammatory response	5,4	1,2E-15
GOTERM_BP_DIRECT	immune response	4,9	1,5E-10
KEGG_PATHWAY	B cell receptor signaling pathway	1,7	6,0E-9
GOTERM_BP_DIRECT	leukocyte migration	2,2	8,0E-9
KEGG_PATHWAY	Cell adhesion molecules (CAMs)	2,3	7,0E-8
GOTERM_BP_DIRECT	positive regulation of T cell proliferation	1,3	1,4E-6
GOTERM_BP_DIRECT	chemotaxis	1,9	2,3E-6
KEGG_PATHWAY	Natural killer cell mediated cytotoxicity	1,9	2,4E-6
GOTERM_BP_DIRECT	type I interferon signaling pathway	1,3	3,3E-6
GOTERM_BP_DIRECT	negative regulation of B cell proliferation	0,6	5,7E-6
KEGG_PATHWAY	Leukocyte transendothelial migration	1,8	1,1E-5
KEGG_PATHWAY	Fc gamma R-mediated phagocytosis	1,5	1,9E-5
KEGG_PATHWAY	Toll-like receptor signaling pathway	1,6	3,8E-5
GOTERM_BP_DIRECT	interferon-gamma-mediated signaling pathway	1,2	5,6E-5
KEGG_PATHWAY	Chemokine signaling pathway	2,2	1,3E-4

Supplementary table 6 | Gene ontology and functional annotation analysis of SC stroma genes not shared with repair SCs

Gene ontology term (GOTERM), cellular compartment (CC), biological process (BP).

Category	Term	% of genes	p-value
UP_KEYWORDS	Endoplasmic reticulum	13,3	2,4E-26
UP_KEYWORDS	Transport	18,9	6,1E-23
GOTERM_CC_DIRECT	endoplasmic reticulum membrane	11,1	2,0E-20
KEGG_PATHWAY	Protein processing in endoplasmic reticulum	4,4	2,1E-17
UP_KEYWORDS	Protein transport	7,8	3,8E-16
UP_KEYWORDS	Acetylation	24,7	1,2E-13
UP_KEYWORDS	Golgi apparatus	8,8	1,2E-13
GOTERM_BP_DIRECT	response to endoplasmic reticulum stress	2,0	6,9E-9
GOTERM_BP_DIRECT	protein folding in endoplasmic reticulum	0,6	3,6E-4
GOTERM_BP_DIRECT	COPII vesicle coating	1,8	9,0E-9
UP_KEYWORDS	Lysosome	3,5	3,6E-8
GOTERM_BP_DIRECT	oxidation-reduction process	6,1	3,4E-7
GOTERM_BP_DIRECT	protein N-linked glycosylation	0,8	3,8E-3

Supplementary table 7 | Gene ontology and functional annotation analysis of injured nerve associated repair SC genes not shared with stromal SCs

Gene ontology term (GOTERM), cellular compartment (CC), biological process (BP).

SUPPLEMENTARY FIGURES AND FIGURE LEGENDS

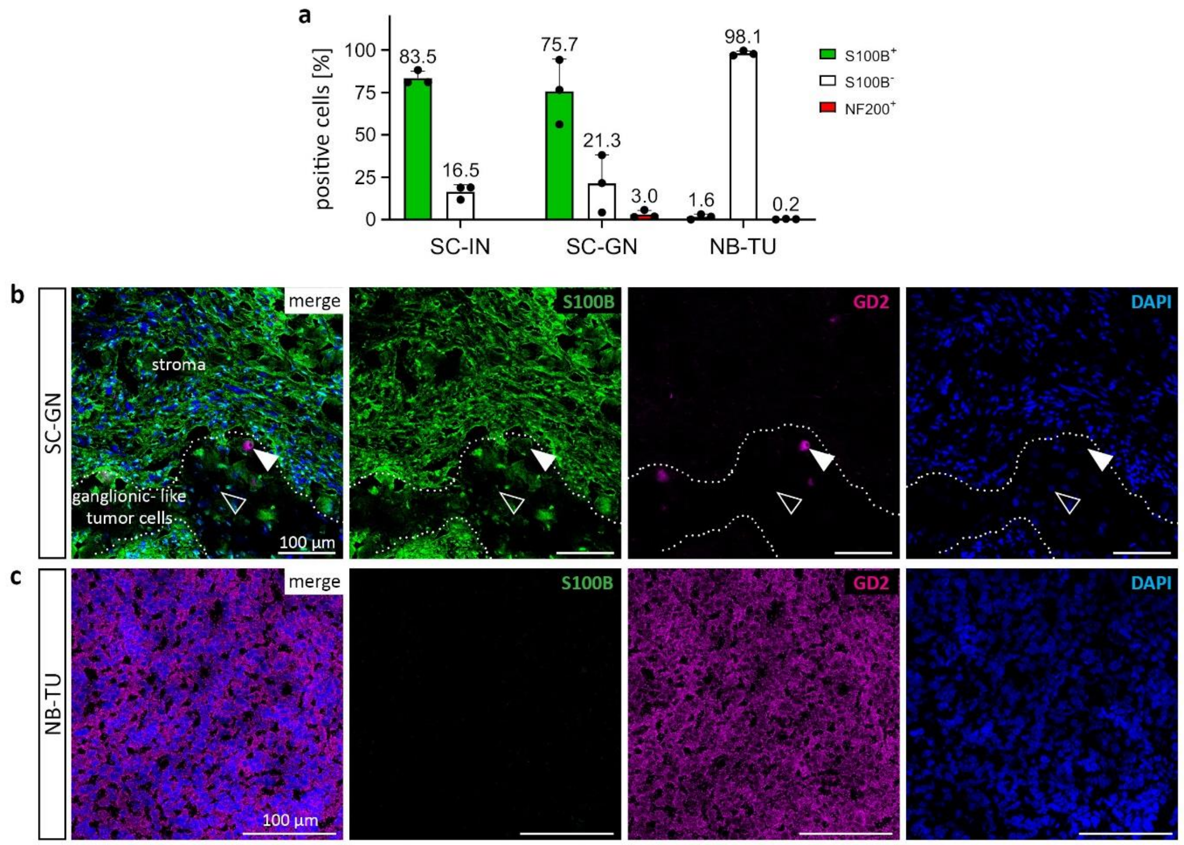

Supplementary Figure 1 | Validation of tissues used for transcriptomic analysis. (a) Quantification of the number of S100B$^+$ SCs and S100B$^-$ cells on injured nerve sections (SC-IN) as well as S100B$^+$ SCs, S100B$^-$ cells, and NF200$^+$ tumor cells in GN (SC-GN) and NB (NB-TU) sections. Data are depicted as mean ± SD (n≥300 cells examined over 3 independent experiments). Representative immunostainings of fresh frozen sections of a GN **(b)** and NB **(c)** stained for S100B, GD2, and DAPI. Filled arrowheads indicate a GD2 positive ganglionic-like tumor cell, lined arrowheads indicate a GD2 negative ganglionic-like tumor cell.

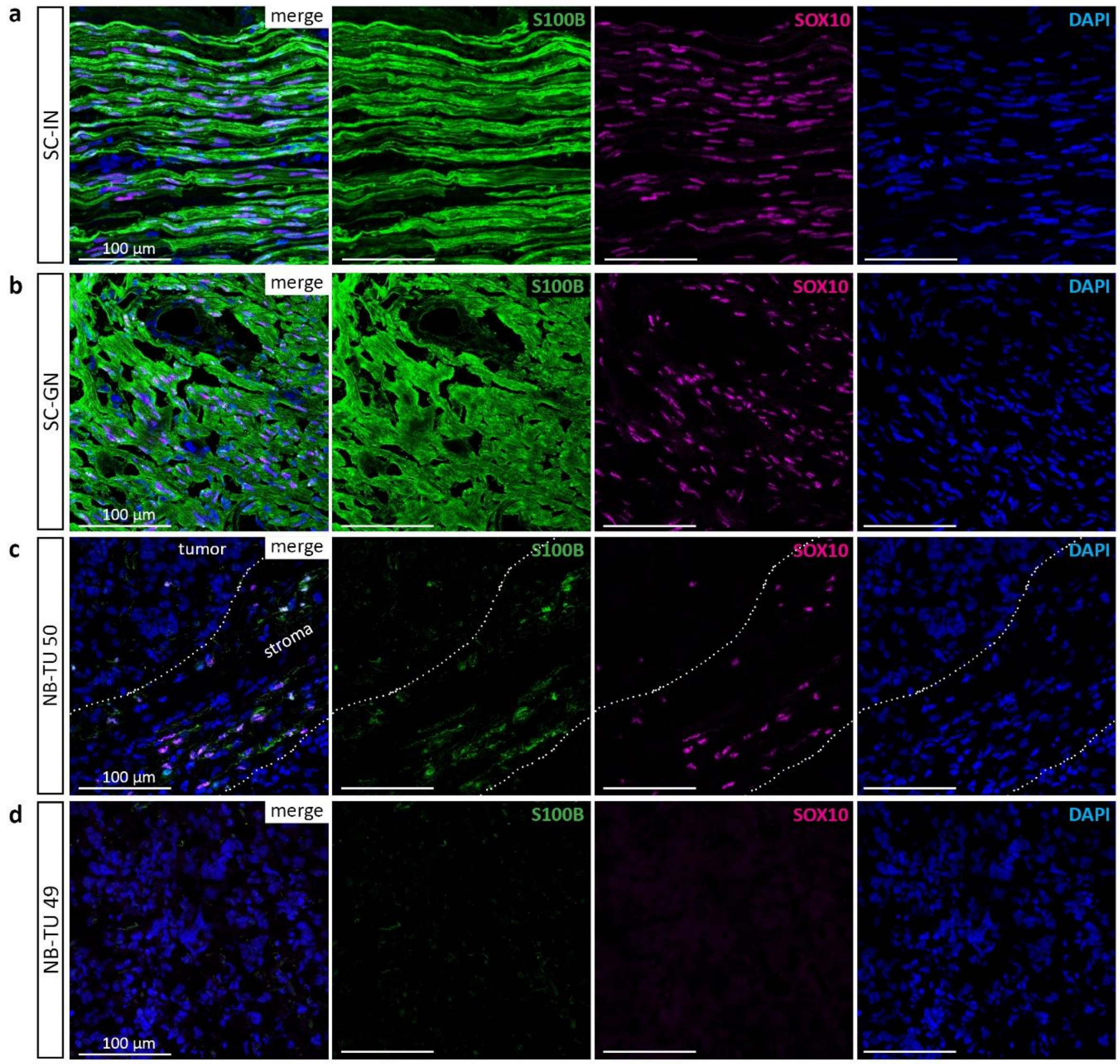

Supplementary Figure 2 | Validation of S100B and SOX10 expression on injured nerve and tumor tissues. Representative immunostainings of fresh frozen sections of an injured nerve **(a)**, a GN **(b)**, and two NBs **(c,d)** stained for S100B, SOX10, and DAPI.

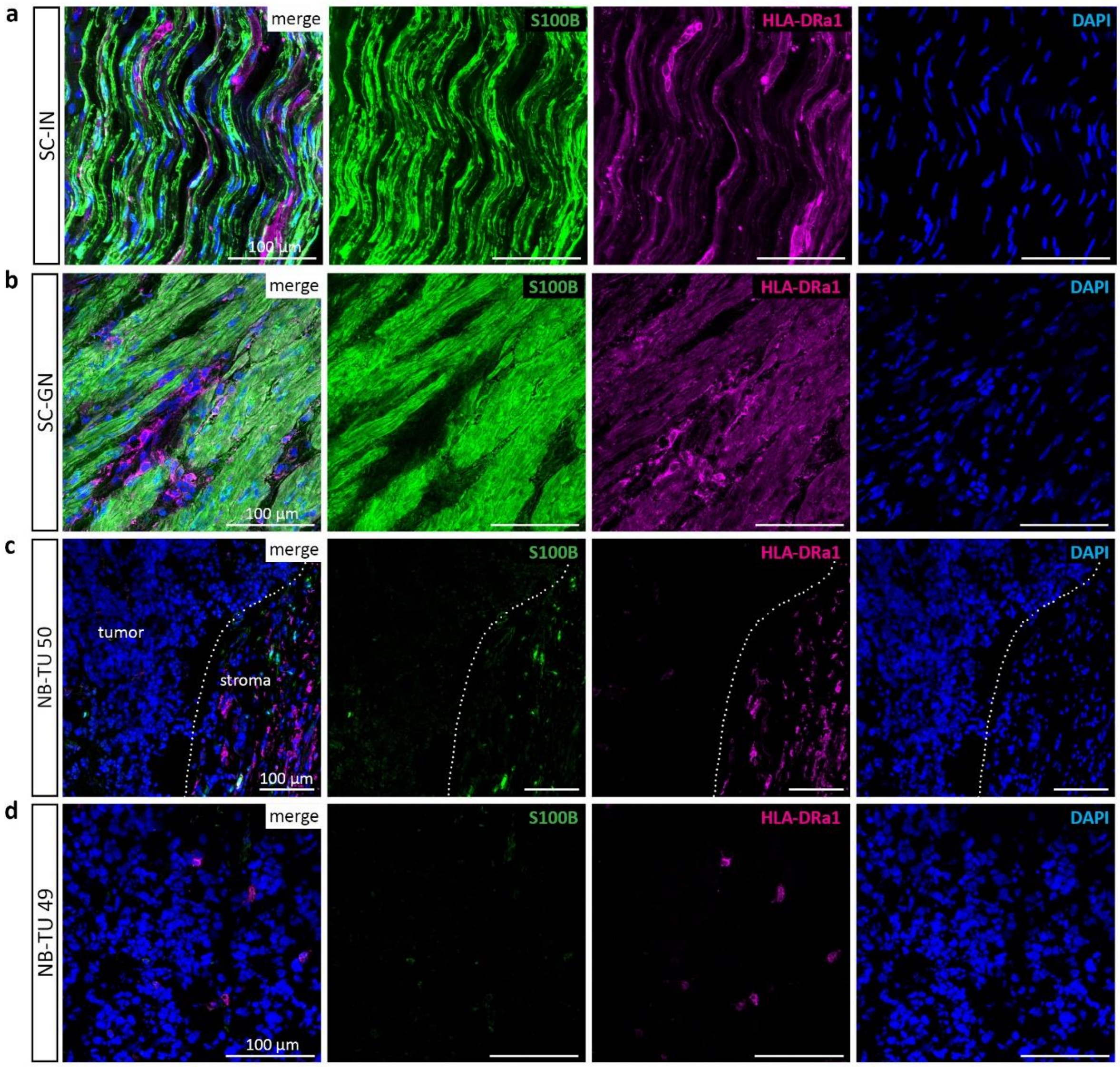

Supplementary Figure 3 | Validation of S100B and HLA-DR expression on injured nerve and tumor tissues. Representative immunofluorescence images of fresh frozen sections of an injured nerve **(a)**, a GN **(b)**, and two NBs **(c,d)** stained for S100B, HLA-DRalpha1, and DAPI.

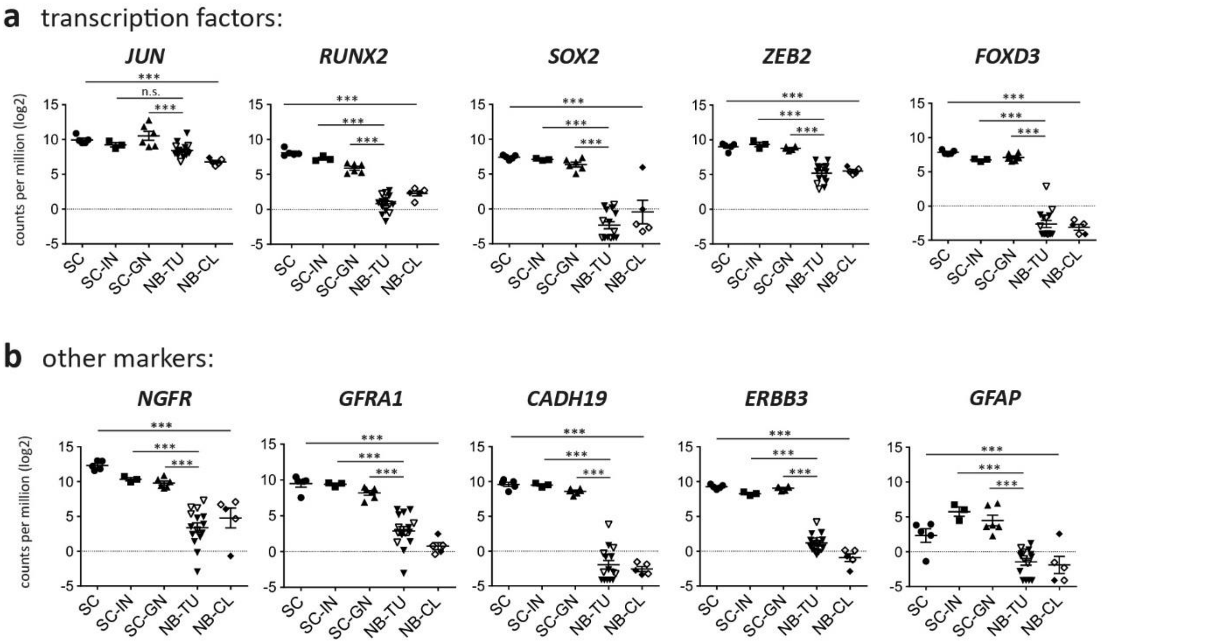

Supplementary Figure 4 | Validation of genes present in developing and dedifferentiated/repair SCs. Expression levels of **(a)** transcription factors and **(b)** other markers associated with developing SCs and dedifferentiated/repair SCs in primary repair-related SCs (SC), injured nerve fascicle tissue (SC-IN), SC stroma rich GN tissue (SC-GN), NB tissue (NB-TU) and NB cell lines (NB-CL). SC: n=5; SC-IN: n=3; SC-GN: n=6; NB-TU: n=15; NB-CL: n=6 biologically independent samples; lined symbols indicate *MYCN* non-amplified non-amplified NB-TUs and NB-CLs. * $p \leq 0.05$, ** $p \leq 0.01$, *** $p \leq 0.001$, n.s. not significant. P-values are given in the Supplementary Data 5, Source data file RNA_seq_cpm_stats_raw; Statistical analysis: differential expression analysis was performed by edgeR and voom as described in the methods section. All p-values were corrected for multiple testing by the Benjamini-Hochberg method. Genes with an adjusted q-value <0.05 and a log2 fold change > 1 (|log2FC|>1) were referred to as significantly regulated. Data are depicted as mean ± SD.

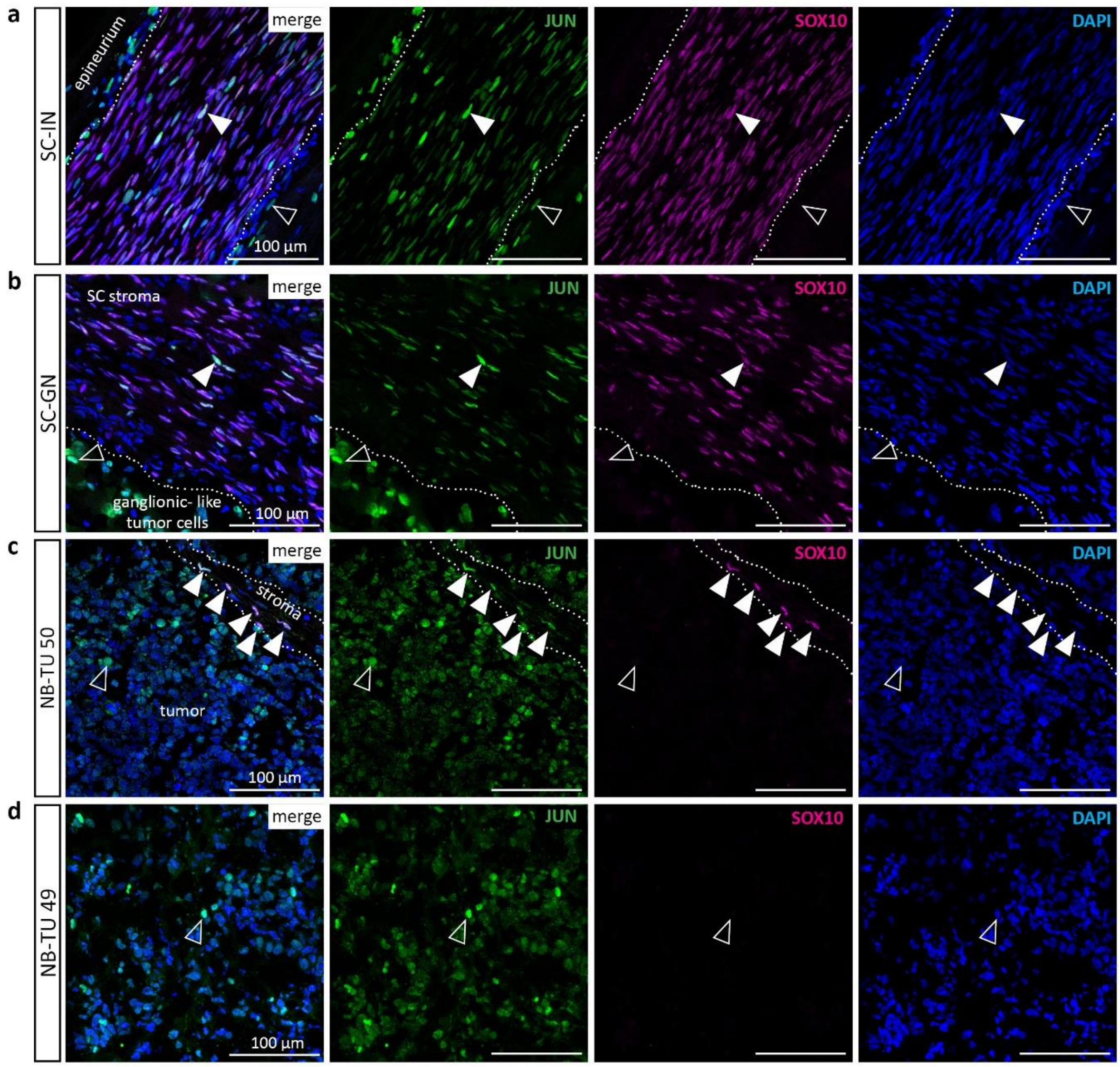

Supplementary Figure 5 | Validation of JUN and SOX10 expression on injured nerve and tumor tissues. Representative immunostainings of fresh frozen sections of an injured nerve **(a)**, a GN **(b)**, and two NBs **(c,d)** stained for JUN, Sox10, and DAPI. Filled arrowheads in (a) indicate JUN$^+$/SOX10$^+$ repair SCs, lined arrowheads in (a) indicate JUN$^+$/SOX10$^-$ epineural cells. Filled arrowheads in (b) indicate JUN$^+$/SOX10$^+$ stromal SCs, lined arrowheads in (b) indicate JUN$^+$/SOX10$^-$ ganglionic-like tumor cells. Filled arrowheads in (c) indicate JUN$^+$/SOX10$^+$ stromal SCs, lined arrowheads in (c) and (d) indicate JUN$^+$/SOX10$^-$ tumor cells.

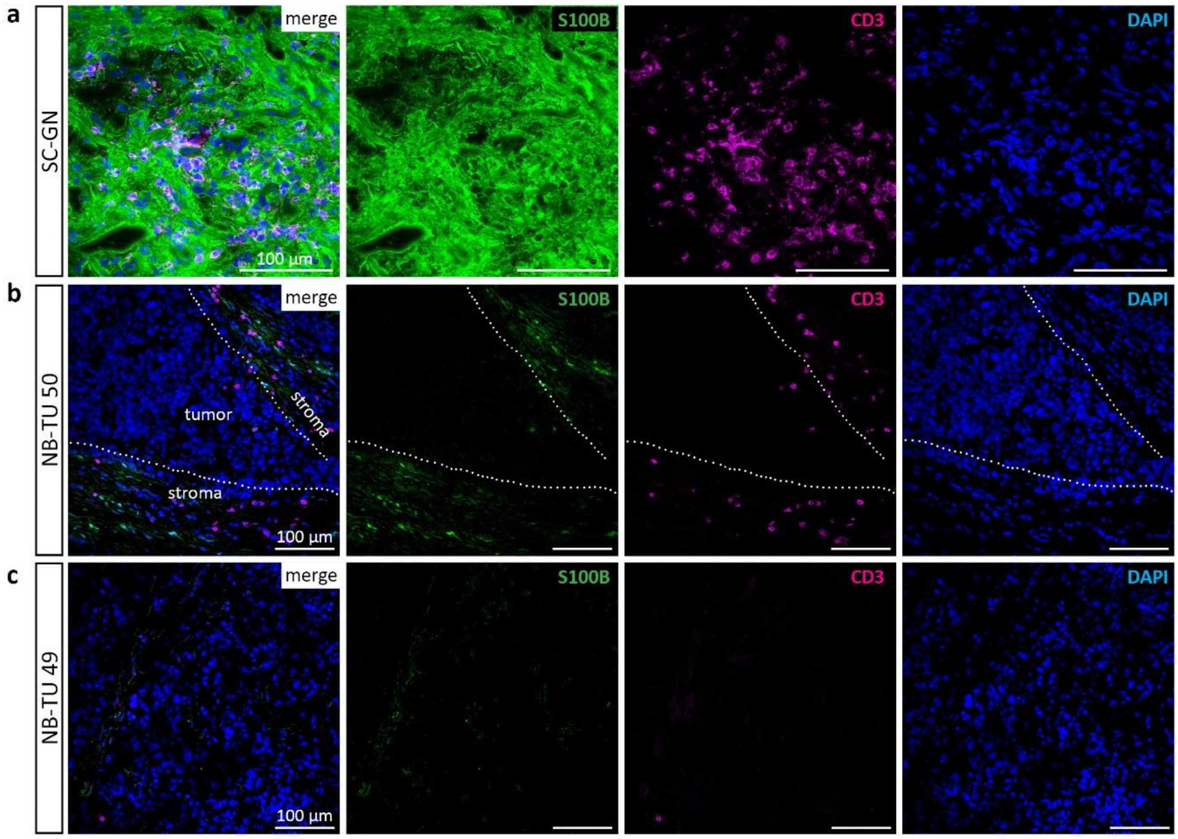

Supplementary Figure 6 | Validation of S100B and CD3 expression on tumor tissues. Representative immunofluorescence images of fresh frozen sections of a GN **(a)**, and two NBs **(b,c)** stained for S100B, CD3, and DAPI.

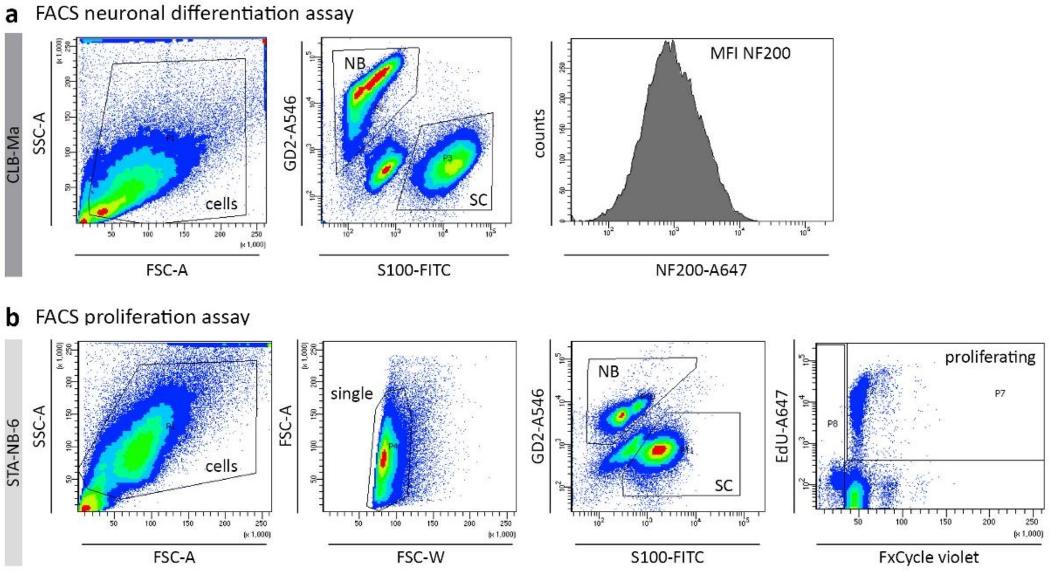

Supplementary Figure 7 | Neuronal differentiation and proliferation analysis of neuroblastoma cell lines in response to repair-related SCs *in vitro*. Detailed FACS gating strategy corresponding to experiments presented in Fig. 4, 5 and 6. **(a)** Neuronal differentiation: 1. FSC/SSC: gating on a defined "cell" population, clearly separate from smaller events. 2. S100-FITC/GD2-A546: gating on a defined, clearly distinct S100 positive GD2 negative "SC" and S100 negative GD2 positive "NB" population. 3. Mean fluorescence intensity of NF200-A647 was determined in the "NB" population. **(b)** Proliferation: 1. FSC/SSC: gating on a defined "cell" population, clearly separate from smaller events. 2. FSC-W/FSC-A: gating on "single" cells. 3. S100-FITC/GD2-A546: gating on a defined, clearly distinct S100 positive GD2 negative "SC" and S100 negative GD2 positive "NB" population. 4. FxCycle Violet/EdU-A647: Within the "NB" cells, the distinct EdU positive cell population was gated and presented as percentage of proliferating cells (excluding the sub-G1 fraction).

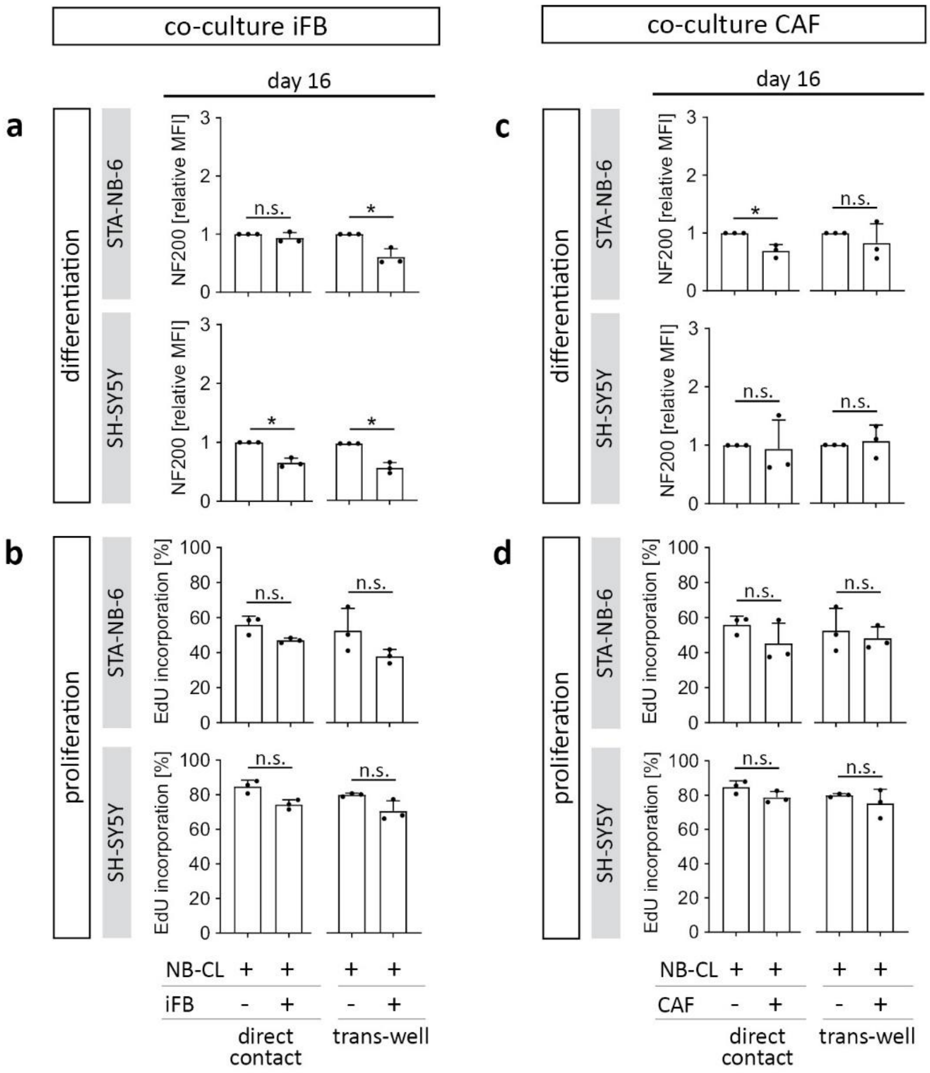

Supplementary Figure 8 | Neuronal differentiation and proliferation analyses of neuroblastoma cell lines in response to immortalized human fibroblasts and cancer-associated fibroblasts *in vitro*. NB cell lines STA-NB-6 and SHSY-5Y were co-cultured with primary immortalized human fibroblasts (iFBs) **(a,b)** and cancer-associated fibroblasts (CAFs) **(c,d)**. Neuronal differentiation (a,c) and proliferation rates (b,c) were analyzed flow cytometry. Bar diagrams show the relative mean fluorescence intensity (MFI) of NF200 ± SD (n=3 independent experiments) in GD2$^+$ NB cell lines upon direct and trans-well co-culture with iFBs (a) (STA-NB-6 trans-well: p=0.04; SH-SY5Y direct: p=0.02 and trans-well: p=0.02) and CAFs (b) (STA-NB-6 direct: p=0.04) at day 16. Bar diagrams show the mean percentage of EdU-incorporation ± SD (n=3 independent experiments) in GD2$^+$ NB cell lines upon direct and trans-well co-culture with iFBs (c) and (CAFs) (d) at day 16; A paired two-tailed Student's t-test comparing against the control was performed. * p ≤ 0.05; n.s. not significant (for all p-values see Source Data file Supplementary Figure 8_raw).

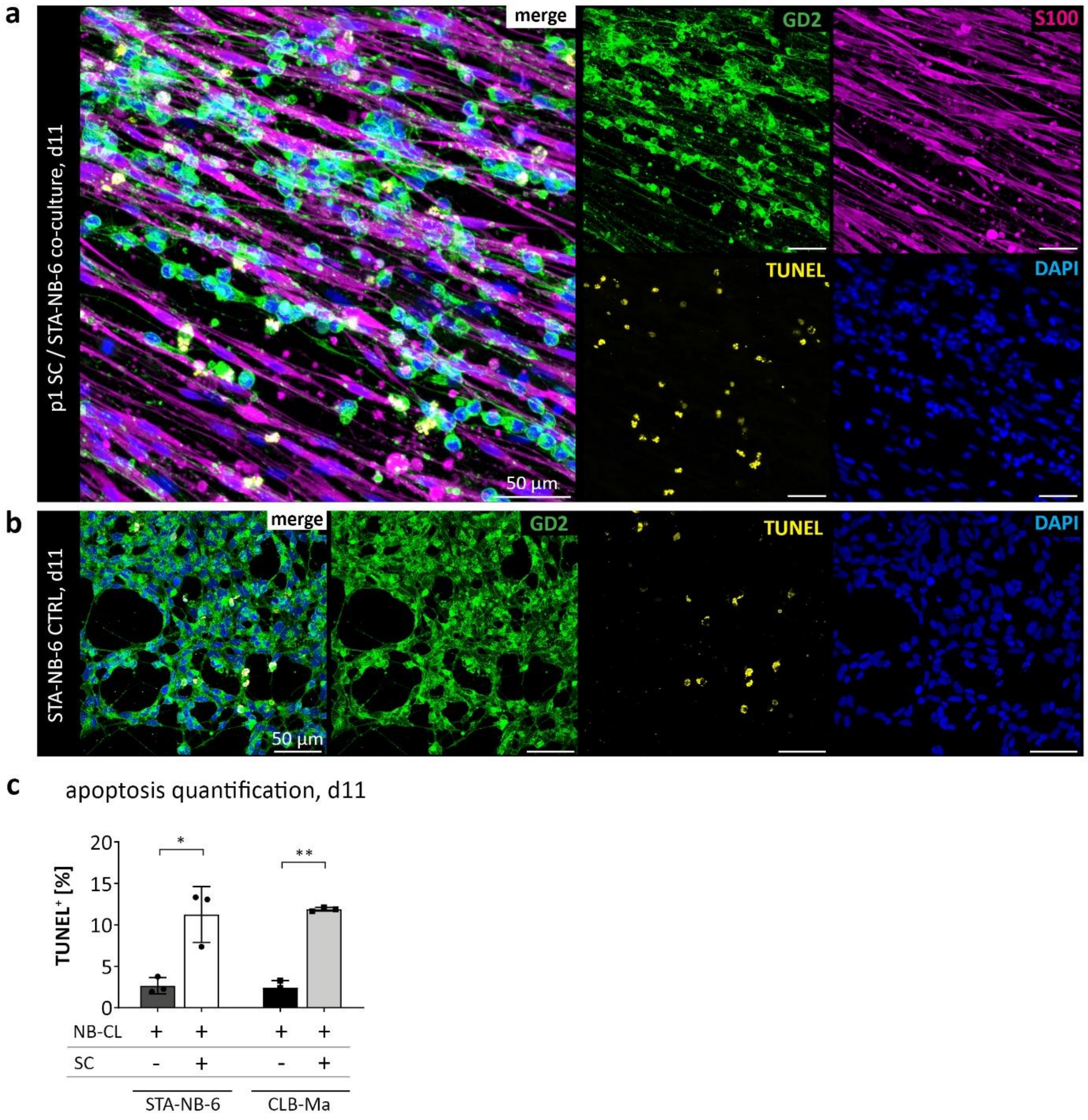

Supplementary Figure 9 | Quantification of apoptotic NB cells after direct contact to repair-related SCs *in vitro*. NB cell lines STA-NB-6 and CLB-Ma were co-cultured with primary SCs in passage 1 (p1) for 11 days followed by immunostainings for GD2, S100B, and DAPI combined with a TUNEL assay that detects fragmented DNA typical for apoptotic cells. Representative immunofluorescence images of STA-NB-6 **(a)** co-cultures and **(b)** control cultures. **(c)** Bar diagrams show the mean percentage of GD2$^+$/TUNEL$^+$ STA-NB-6 and CLB-Ma cells in control and co-cultures ± SD (788 – 2797 cells quantified per condition and replicate, n=3 independent experiments) at day 11; A paired two-tailed Student's t-test comparing against the control NB-CL was performed. STA-NB-6: $p=0.043$; CLB-Ma: $p=0.004$; * $p \leq 0.05$; ** $p \leq 0.01$.

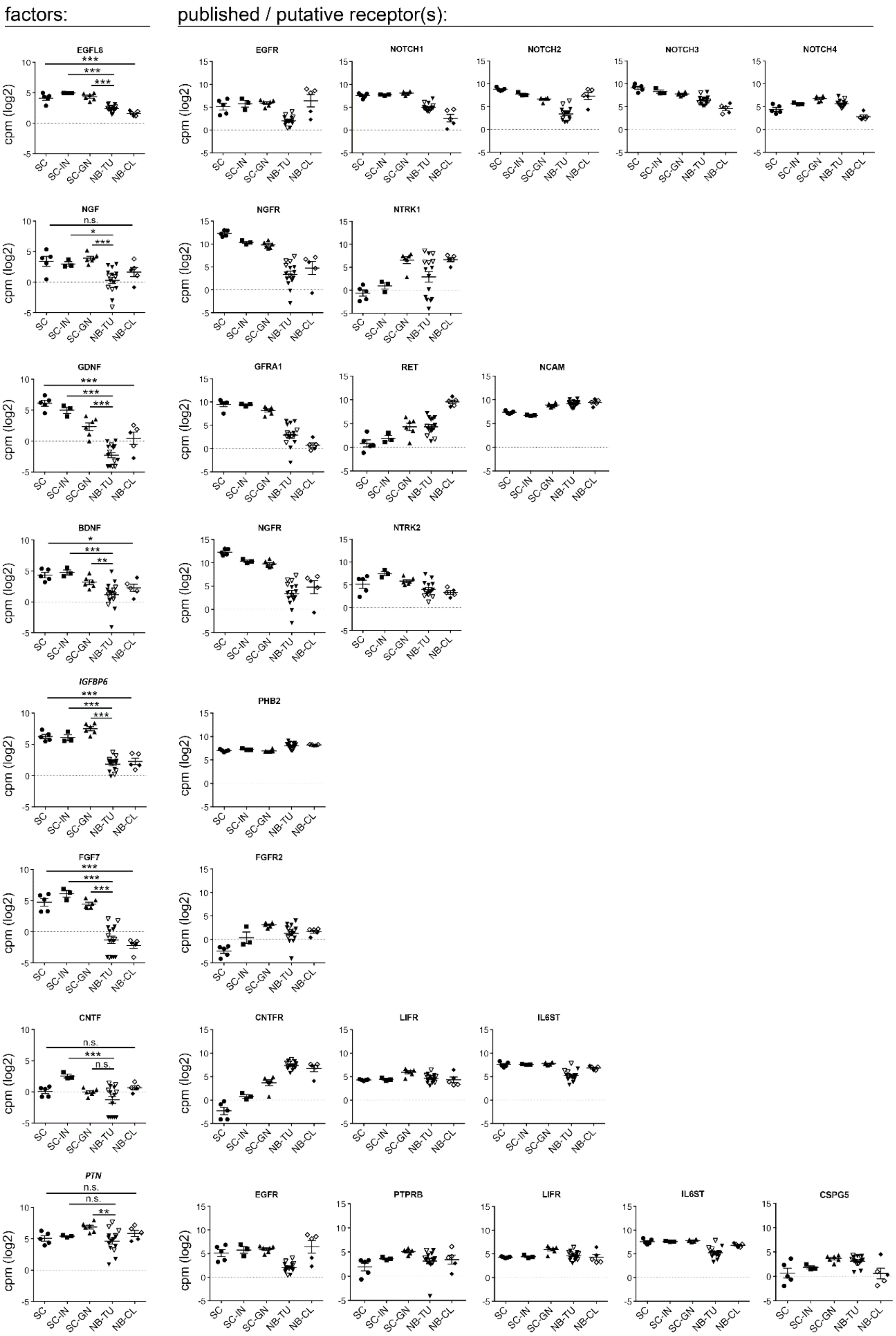

Supplementary Figure 10 | RNA-seq results for secreted candidate factors and their (putative) receptors. Expression levels of chosen candidate factors *NGF*, *EGFL8*, *BDNF*, *GDNF*, *IGFBP6*, *FGF7*, *CNTF* and *PTN* and their

(putative) receptors in primary repair-related SCs (SC), injured nerve fascicle tissue (SC-IN), SC stroma rich GN tissue (SC-GN), NB tissue (NB-TU) and NB cell lines (NB-CL); SC: n=5; SC-IN: n=3; SC-GN: n=6; NB-TU: n=18; NB-CL: n=6 biologically independent samples; lined symbols indicate *MYCN* non-amplified non-amplified NB-TUs and NB-CLs. * p ≤ 0.05, ** p ≤ 0.01, *** p ≤ 0.001, n.s. not significant. P-values are given in the Source Data file RNA_seq_cpm_stats_raw; Statistical analysis: differential expression analysis was performed by edgeR and voom as described in the methods section. All p-values were corrected for multiple testing by the Benjamini-Hochberg method. Genes with an adjusted q-value <0.05 and a log2 fold change > 1 (|log2FC|>1) were referred to as significantly regulated. Data are depicted as mean ± SD.

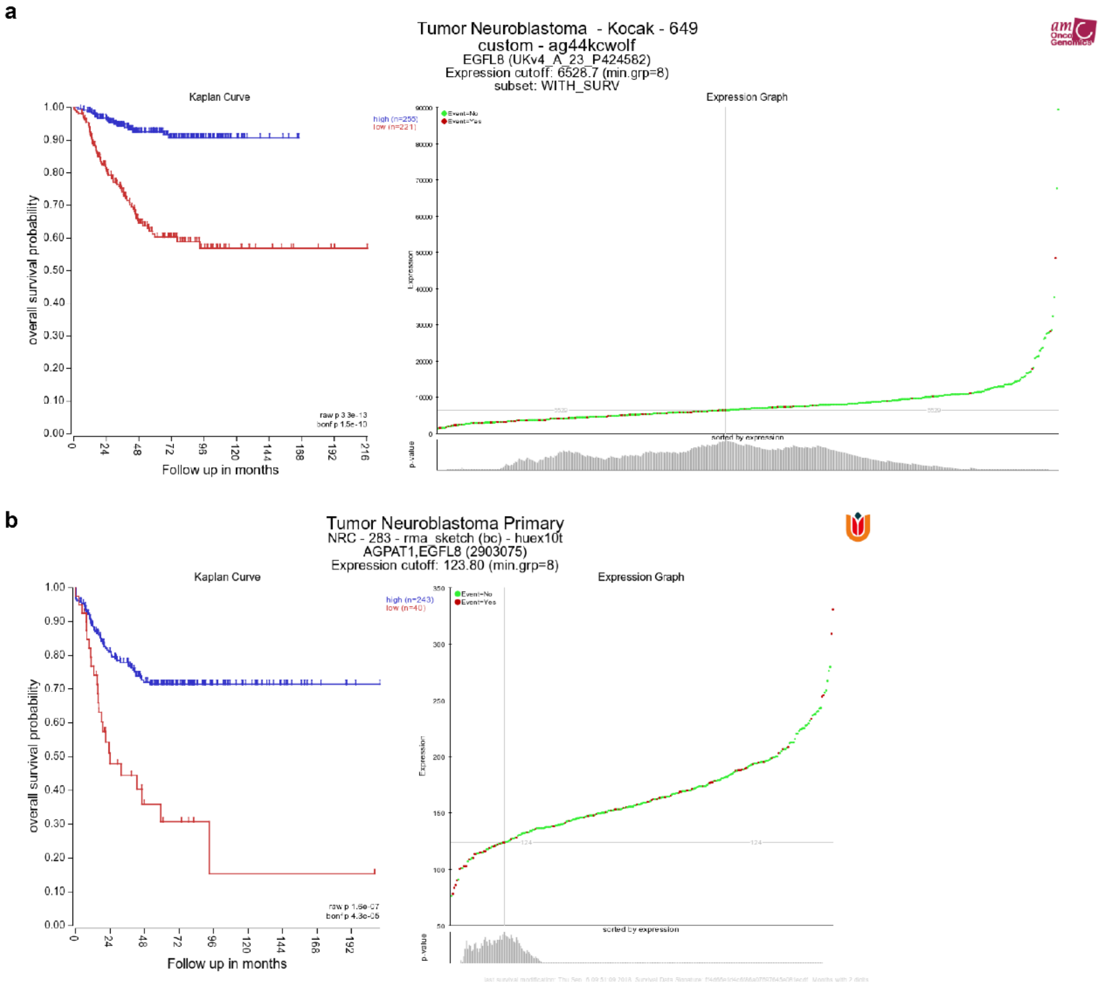

Supplementary Figure 11 | *EGFL8* expression in peripheral neuroblastic tumors. One gene view for EGFL8 using the R2 Genomics Analysis and Visualization platform (https://r2.amc.nl) for the Kocak dataset (GSE45547) containing 649 tumor specimens **(a)** and the NRC dataset (GSE85047) containing 283 tumor specimen **(b).** Kaplan Curves of the overall survival probability of patients with available survival data according to EGFL8 high and EGFL8 low expressing tumors and the corresponding expression graphs are shown.

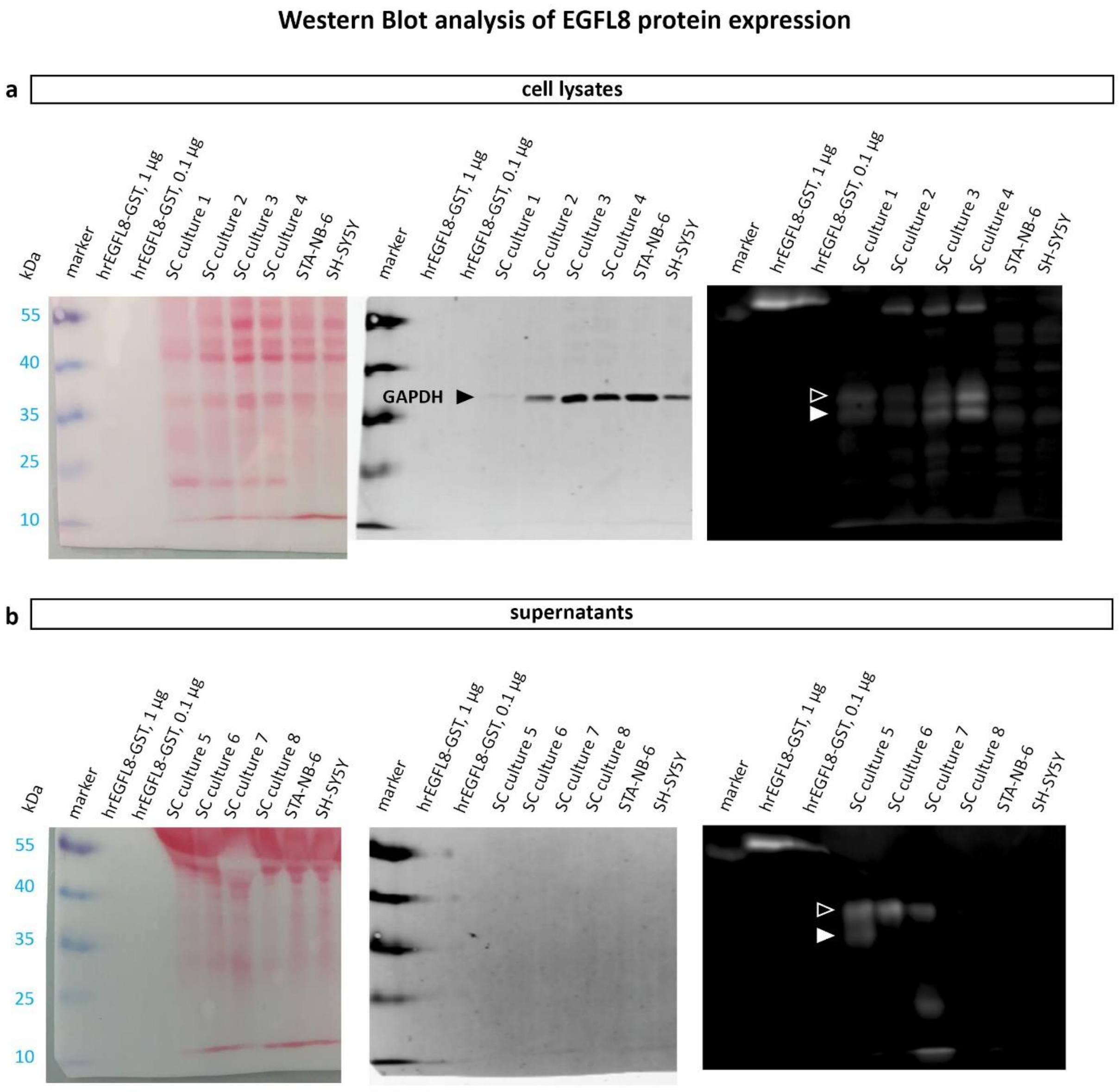

Supplementary Figure 12 | EGFL8 protein expression in cell lysates and supernatants. Ponceau staining, chemiluminescence detection of GAPDH (loading control) and immunofluorescence detection of EGFL8 are shown for SC and NB cell lysates **(a)** and supernatants **(b)**. Filled white arrowheads indicate bands of about 32 kDa, the proposed molecular weight of EGFL8, lined white arrowheads indicate bands of about 37 kDa that could represent the EGFL8 protein with posttranscriptional modifications. Human recombinant (hr) EGFL8 (32 kDa) with a GST-tag (26 kDa) was used as positive control.

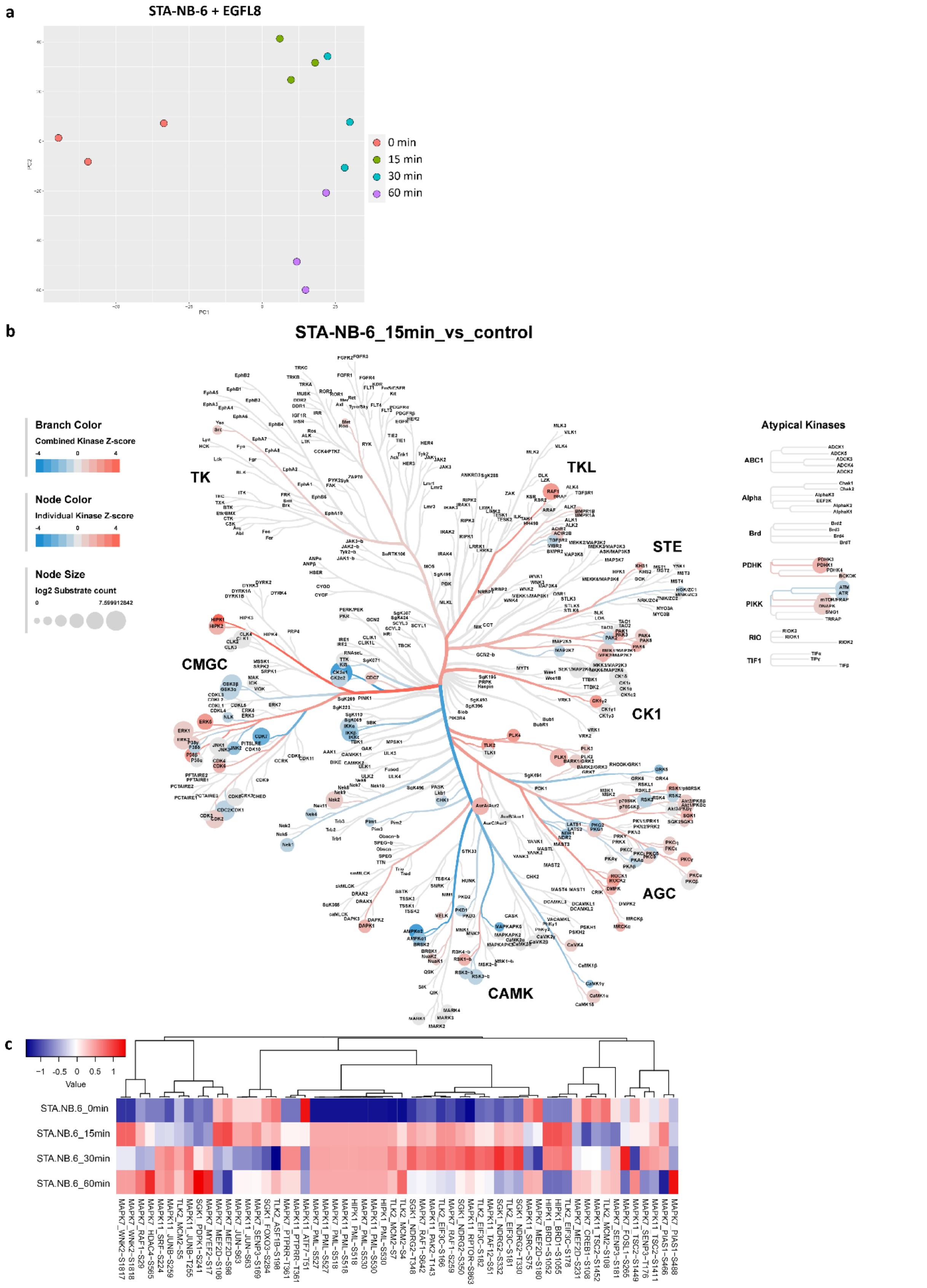

Supplementary Figure 13 | EGFL8 induced kinome activation in STA-NB-6 cells. EGFL8 responsive STA-NB-6 NB primary cultures were left untreated (0 min) or induced with 100 ng/mL EGFL8 for 15, 30 or 60 min (n=3

independent biological replicates). LC-MS/MS was performed following phospho-peptide enrichment. **(a)** PCA analysis of detected phosphopeptides. **(b)** Kinases identified by KSEA upon 15 min EGFL8 versus untreated control projected onto the Kinome Tree [8]. Branch color and node color correspond to combined and individual enrichment z-scores, respectively. Node size corresponds to the log2 kinase substrate counts. Kinases without phosphorylated substrates are not represented by nodes. **(c)** heatmap depicts z-scores of phosphorylated substrates. X-axis labels correspond to kinase gene, substrate gene and amino acid position of phospho-modification. This Figure is related to **Figure 7**.

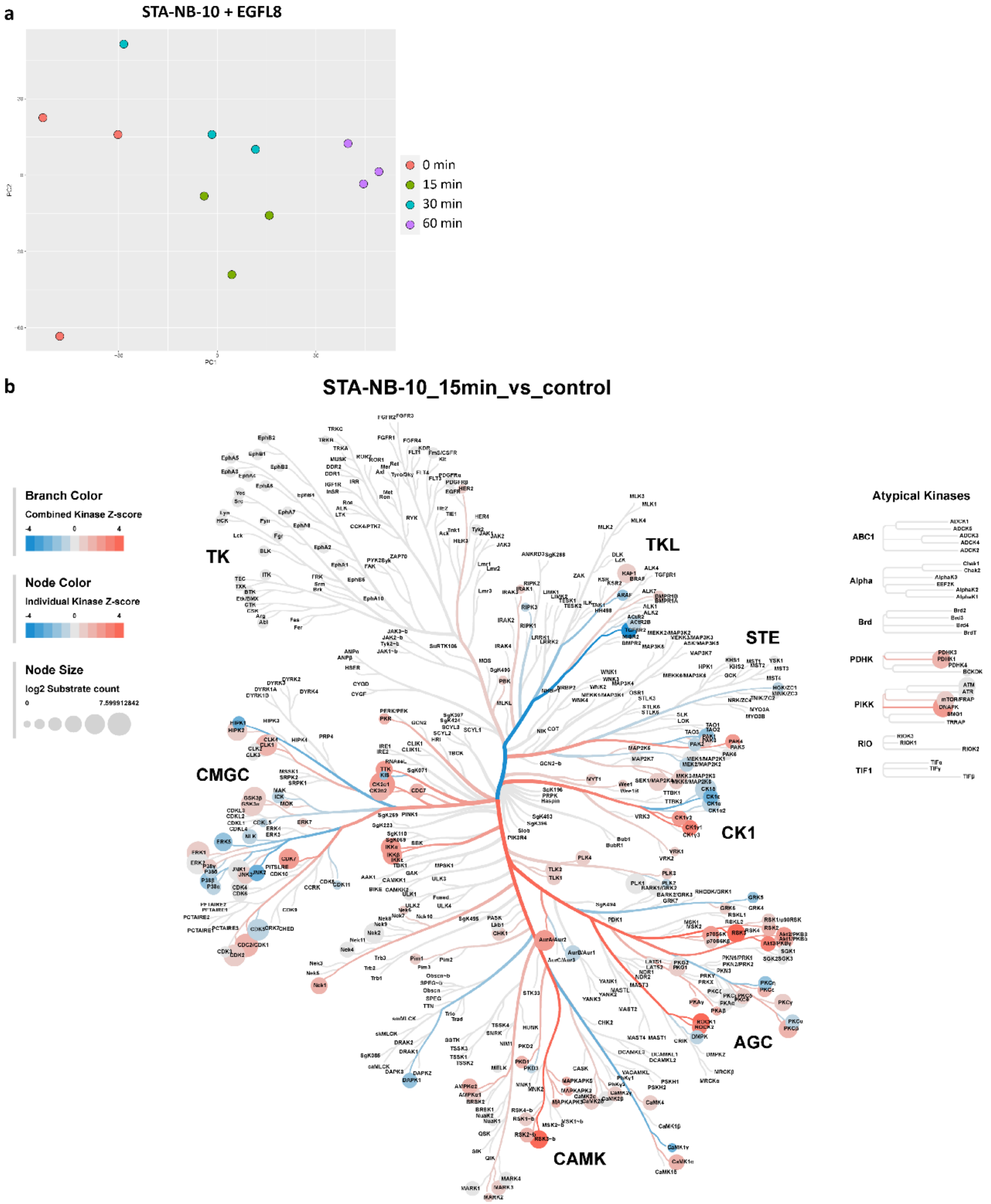

Supplementary Figure 14 | EGFL8 induced kinome activation in STA-NB-10 cells. EGFL8 non-responsive STA-NB-10 NB primary cultures were left untreated (0 min) or induced with 100 ng/mL EGFL8 for 15, 30 or 60 min (n=3 independent biological replicates). LC-MS/MS was performed following phospho-peptide enrichment. **(a)** PCA analysis of detected phosphopeptides. **(b)** Kinases identified by KSEA upon 15 min EGFL8 versus untreated control projected onto the Kinome Tree [8]. Branch color and node color correspond to combined and individual enrichment z-scores, respectively. Node size corresponds to the log2 kinase substrate counts. Kinases without phosphorylated substrates are not represented by nodes. This Figure is related to **Figure 7**.

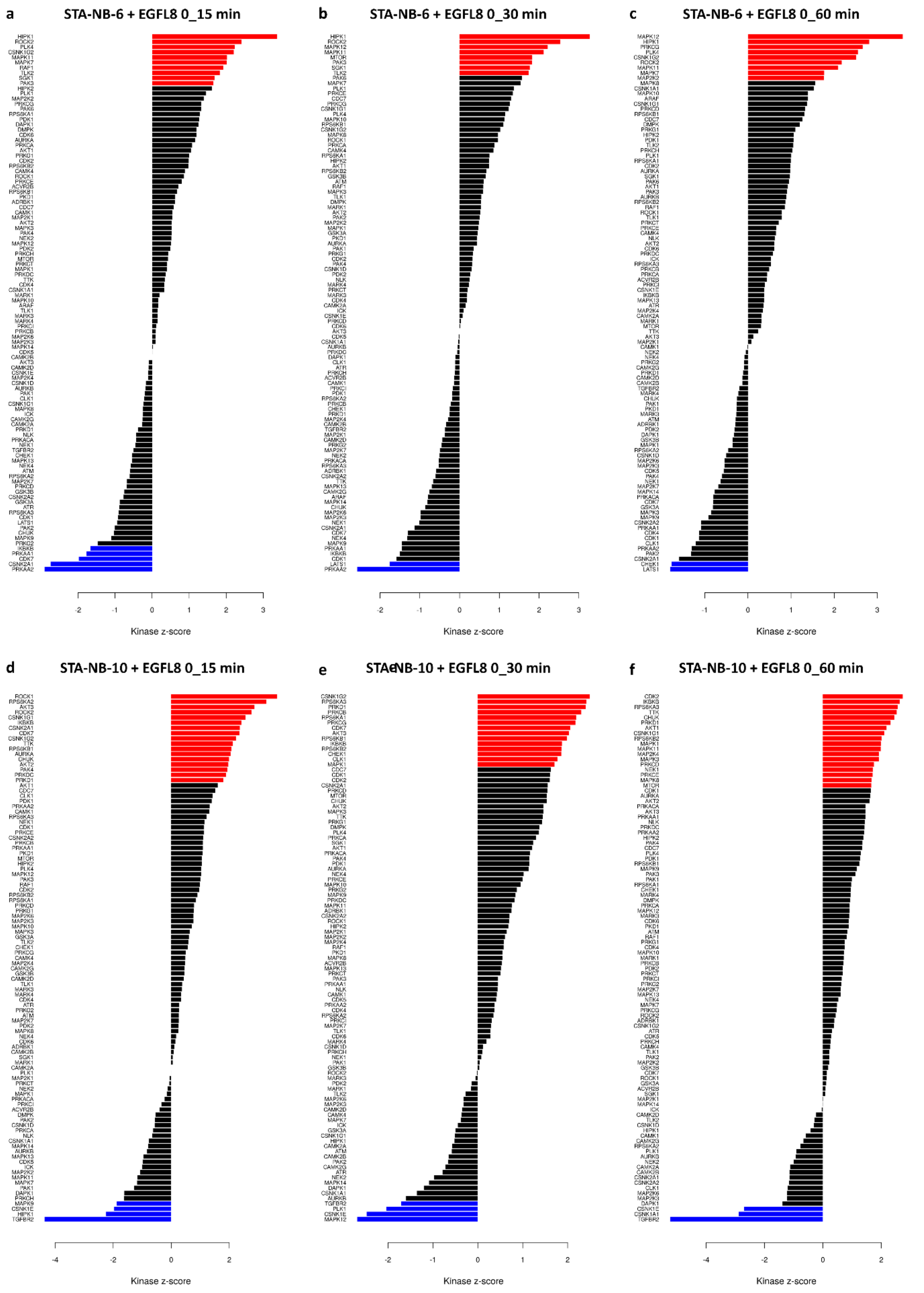

Supplementary Figure 15 | EGFL8 induced kinome activation in STA-NB-6 and STA-NB-10 cells. NB primary cultures were left untreated (0 min) or induced with 100 ng/mL EGFL8 for 15, 30 or 60 min (n=3 independent

biological replicates). LC-MS/MS was performed following phospho-peptide enrichment. Waterfall plots depict kinases identified by kinase-substrate enrichment analysis (KSEA) (cut-off ≥ 3 substrates; z-score ≥ 1; p ≤ 0.05). red bars represent significantly enriched kinases; blue bars de-enriched kineases. Exact p-values are given in supplementary data 3. Statistical test used for data presented in this figure and supplementary data 2: KSEA of class 1 phosphosites (p > 0.75) was performed using PhosphoSitePlus and NetworKIN as described in the methods section.

REFERENCES

1. Combaret V*, et al.* Sensitive detection of numerical and structural aberrations of chromosome 1 in neuroblastoma by interphase fluorescence in situ hybridization. Comparison with restriction fragment length polymorphism and conventional cytogenetic analyses. *Int J Cancer* **61**, 185-191 (1995).

2. Fischer M, Berthold F. Characterization of the gene expression profile of neuroblastoma cell line IMR-5 using serial analysis of gene expression. *Cancer Lett* **190**, 79-87 (2003).

3. Momoi M, Kennett RH, Glick MC. A membrane glycoprotein from human neuroblastoma cells isolated with the use of a monoclonal antibody. *J Biol Chem* **255**, 11914-11921 (1980).

4. Biedler JL, Helson L, Spengler BA. Morphology and growth, tumorigenicity, and cytogenetics of human neuroblastoma cells in continuous culture. *Cancer Res* **33**, 2643-2652 (1973).

5. Biedler JL, Roffler-Tarlov S, Schachner M, Freedman LS. Multiple neurotransmitter synthesis by human neuroblastoma cell lines and clones. *Cancer Res* **38**, 3751-3757 (1978).

6. Ambros IM*, et al.* Neuroblastoma cells can actively eliminate supernumerary MYCN gene copies by micronucleus formation--sign of tumour cell revertance? *Eur J Cancer* **33**, 2043-2049 (1997).

7. Stock C*, et al.* Genes proximal and distal to MYCN are highly expressed in human neuroblastoma as visualized by comparative expressed sequence hybridization. *Am J Pathol* **172**, 203-214 (2008).

8. Metz KS*, et al.* Coral: Clear and Customizable Visualization of Human Kinome Data. *Cell Systems* **7**, 347-350.e341 (2018).
