# Supplementary material for: Schwann cell plasticity regulates neuroblastic tumor cell differentiation via epidermal growth factor-like protein 8
Source: Nat Commun. 2021 Mar 12;12:1624. doi: 10.1038/s41467-021-21859-0 (PMC7954855; doi:10.1038/s41467-021-21859-0)
Supplement: Supplementary file 3 — Description of Additional Supplementary Files [file 41467_2021_21859_MOESM3_ESM.docx]

**Supplementary Movie 1.** Representative immunostaining of a repair-related SC culture stained for NGFR, EGFL8, and DAPI. The video of z-stacks shows the SC body with membranous NGFR staining and intracellular EGFL8 signals within vesicle-like structures.

**Supplementary Data 1: Global proteomics data STA-NB-6 and STA-NB-10.** EGFL8 responsive STA-NB-6 and non-responsive STA-NB-10 NB primary cultures were left untreated (0 min) or induced with 100 ng/mL EGFL8 for 15, 30 or 60 min. LC-MS/MS was performed.

**Supplementary Data 2: Phosphoproteomics data STA-NB-6 and STA-NB-10.** EGFL8 responsive STA-NB-6 and non-responsive STA-NB-10 NB primary cultures were left untreated (0 min) or induced with 100 ng/mL EGFL8 for 15, 30 or 60 min. LC-MS/MS was performed following phospho-peptide enrichment.

**Supplementary Data 3: Kinase substrate enrichment analysis (KSEA) Kinase Scores.** EGFL8 responsive STA-NB-6 and non-responsive STA-NB-10 NB primary cultures were left untreated (0 min) or induced with 100 ng/mL EGFL8 for 15, 30 or 60 min. LC-MS/MS was performed following phospho-peptide enrichment. Statistical test: KSEA of class 1 phosphosites (p > 0.75) was performed using PhosphoSitePlus and NetworKIN applying a NetworkKIN score cutoff of 2, p-value cutoff of 0.05 and substrate count cutoff 3 as described in the methods section.

**Supplementary Data 4: Kinase substrate enrichment analysis (KSEA) Kinase Substrates.** EGFL8 responsive STA-NB-6 and non-responsive STA-NB-10 NB primary cultures were left untreated (0 min) or induced with 100 ng/mL EGFL8 for 15, 30 or 60 min. LC-MS/MS was performed following phospho-peptide enrichment. Statistical test: KSEA of class 1 phosphosites (p > 0.75) was performed using PhosphoSitePlus and NetworKIN applying a NetworkKIN score cutoff of 2, p-value cutoff of 0.05 and substrate count cutoff 3 as described in the methods section.
