# Supplementary material for: Schwann cell plasticity regulates neuroblastic tumor cell differentiation via epidermal growth factor-like protein 8
Source: Nat Commun. 2021 Mar 12;12:1624. doi: 10.1038/s41467-021-21859-0 (PMC7954855; doi:10.1038/s41467-021-21859-0)
Supplement: Supplementary file 9 — Reporting Summary [file 41467_2021_21859_MOESM9_ESM.pdf]

nature research

Corresponding author(s): Taschner-Mandl Sabine

Last updated by author(s): Jan 13, 2021

Reporting Summary

Nature Research wishes to improve the reproducibility of the work that we publish. This form provides structure for consistency and transparency in reporting. For further information on Nature Research policies, see Authors & Referees and the Editorial Policy Checklist .

Statistics

For all statistical analyses, confirm that the following items are present in the figure legend, table legend, main text, or Methods section.

n/a	Confirmed	
☐	☒	The exact sample size (*n*) for each experimental group/condition, given as a discrete number and unit of measurement
☐	☒	A statement on whether measurements were taken from distinct samples or whether the same sample was measured repeatedly
☐	☒	The statistical test(s) used AND whether they are one- or two-sided<br>*Only common tests should be described solely by name; describe more complex techniques in the Methods section.*
☒	☐	A description of all covariates tested
☐	☒	A description of any assumptions or corrections, such as tests of normality and adjustment for multiple comparisons
☐	☒	A full description of the statistical parameters including central tendency (e.g. means) or other basic estimates (e.g. regression coefficient) AND variation (e.g. standard deviation) or associated estimates of uncertainty (e.g. confidence intervals)
☐	☒	For null hypothesis testing, the test statistic (e.g. *F*, *t*, *r*) with confidence intervals, effect sizes, degrees of freedom and *P* value noted<br>*Give P values as exact values whenever suitable.*
☒	☐	For Bayesian analysis, information on the choice of priors and Markov chain Monte Carlo settings
☒	☐	For hierarchical and complex designs, identification of the appropriate level for tests and full reporting of outcomes
☒	☐	Estimates of effect sizes (e.g. Cohen's *d*, Pearson's *r*), indicating how they were calculated

Our web collection on statistics for biologists contains articles on many of the points above.

Software and code

Policy information about availability of computer code

Data collection

FACS data were acquired using the FACSDiva software version 8.0 (Becton Dickinson).
For confocal microscopy images were acquired using the Leica Leica application suite X version 1.8.1.13759 (Leica).
Phase contrast microscopy images were generated using a Zeiss Axiovert 40C with the pixelink application version AL/A6XX.
Western Blot images were acquired using the Odyssey software version 3.0 (Odyssey).
The bulk RNA sequencing experiments were sequenced using an Illumina HiSeq2000 sequencer with corresponding Illumina HiSeq Control software version 2.2.38, RTA version 1.18.61, HiSeq serial number HWI-ST999; basecalling was done with Illumina bcl2fastq-1.8.4.
Liquid chromatography-mass spectrometry: LC-MS/MS analyses were performed using a Dionex Ultimate 3000 nano LC-system coupled to a QExactive orbitrap mass spectrometer with software TUNE version 2.5-204201/2.5.0.2042 and Chromeleon version 6.0 (all Thermo Fisher Scientific).

Data analysis

FACS analysis: FACSDiva software version 8.0 (Becton Dickinson).
Confocal microscopy: images were processed using the Leica application suite X version 1.8.1.13759 or LAS AF Lite software 4.0 (Leica).
RNA-sequencing: Short read sequencing data was quality checked using FASTQC v0.11.5 (http://www.bioinformatics.babraham.ac.uk/projects/fastqc) and QoRTs [Hartley, 2015] and then aligned to the human genome hs37d5 (ftp://ftp.1000genomes.ebi.ac.uk/) using the STAR aligner v2.5.3a [Dobin, 2013] yielding a minimum of 11.6 million aligned reads in each sample. Further analysis was performed in R v3.4.1 statistical environment using Bioconductor v3.5 packages [Gentleman, 2004]. Count statistics for Ensembl (GRCh37.75) genes were obtained by the "featureCounts" function (package "Rsubread") and differential expression analysis was performed by edgeR and voom [Law, 2014;Ritchie, 2015]. For differential gene expression analysis only genes passing a cpm (counts per gene per million reads in library) cut-off of 1 in more than two samples were included. All p-values were corrected for multiple testing by the Benjamini-Hochberg method. Genes with an adjusted q-value <0.05 and a log2 fold change > 1 (|log2FC|>1) were referred to as 'significantly regulated' and used for functional annotation analysis via DAVID database [Huang da, 2009].
Liquid chromatography-mass spectrometry: For the identification and label free quantification of proteins, the MaxQuant software package (version 1.6.1.0) [1] was used essentially as previously described [2]. The human UniProt database (version 03/2018, restricted to reviewed entries only) with 20316 entries was used for the search, and the false discovery rate (FDR) was set to 0.01 on both peptide

and protein level. The alignment time window was set to 1 min, with a match time window of 5 min. The four data matrices obtained as described above were loaded into Perseus software (version 1.6.7.0), followed by filtering those analytes that were present in at least 70% of samples in at least one group [3]. Next, data were log 2 transformed, and missing values were replaced by normally distributed random numbers with a set width of 0.3 and a downshift of 1.8. A two sided t-test was applied for statistical significance testing with number of randomizations set to 250, the FDR threshold was set to 0.05 and the S0 value to 0.1.

[1] J. Cox, M. Mann, MaxQuant enables high peptide identification rates, individualized p.p.b.-range mass accuracies and proteome-wide protein quantification, Nat Biotechnol 26(12) (2008) 1367-72.
[2] B. Neuditschko, L. Janker, L. Niederstaetter, J. Brunmair, K.
Krivanek, S. Izraely, O. Sagi-Assif, T. Meshel, B. Keppler, G. Del Favero, I.P. Witz, C. Gerner, The challenge of classifying metastatic cell properties by molecular profiling exemplified with cutaneous melanoma cells and their cerebral metastasis from patient derived mouse xenografts, Mol Cell Proteomics (2019).
[3] S. Tyanova, T. Temu, P. Sinitcyn, A. Carlson, M.Y. Hein, T. Geiger, M. Mann, J. Cox, The Perseus computational platform for comprehensive analysis of (prote)omics data, Nature methods 13(9) (2016) 731–740.

Statistical analysis: GraphPad Prism version 5. Microsoft Excel 2016, ImageJ v2.1.0 plugin NeuronJ v1.4.3.

For manuscripts utilizing custom algorithms or software that are central to the research but not yet described in published literature, software must be made available to editors/reviewers. We strongly encourage code deposition in a community repository (e.g. GitHub). See the Nature Research guidelines for submitting code & software for further information.

Data

Policy information about availability of data

All manuscripts must include a data availability statement. This statement should provide the following information, where applicable:

- Accession codes, unique identifiers, or web links for publicly available datasets
- A list of figures that have associated raw data
- A description of any restrictions on data availability

All data sets produced and used in this study are available in public repositories as listed in S.Table 4. RNA-sequencing datasets were uploaded to the gene expression omnibus (GEO) repository (https://www.ncbi.nlm.nih.gov/geo/) with the dataset identifiers GSE90711 (https://www.ncbi.nlm.nih.gov/geo/query/acc.cgi?acc=GSE90711), GSE94035 (https://www.ncbi.nlm.nih.gov/geo/query/acc.cgi?acc=GSE94035), GSE147635 (https://www.ncbi.nlm.nih.gov/geo/query/acc.cgi?acc=GSE147635), the Kocak dataset GSE45547 (https://www.ncbi.nlm.nih.gov/geo/query/acc.cgi?acc=GSE45547) and NRC dataset GSE85047 (https://www.ncbi.nlm.nih.gov/geo/query/acc.cgi?acc=GSE85047) are publicly available. The mass spectrometry global and phosphospho-proteomics data have been deposited to the ProteomeXchange Consortium (http://proteomecentral.proteomexchange.org) via the PRIDE partner repository 101 with the dataset identifier PXD018267 (https://www.ebi.ac.uk/pride/archive/projects/PXD018267) and PXD022217 (https://www.ebi.ac.uk/pride/archive/projects/PXD022217) and are publicly available. Source data and a list of figures that have associated raw data is available in S.Data 4.

Field-specific reporting

Please select the one below that is the best fit for your research. If you are not sure, read the appropriate sections before making your selection.

☒ Life sciences ☐ Behavioural & social sciences ☐ Ecological, evolutionary & environmental sciences

For a reference copy of the document with all sections, see nature.com/documents/nr-reporting-summary-flat.pdf

Life sciences study design

All studies must disclose on these points even when the disclosure is negative.

Sample size	Sample size was determined based on previous experience with transcriptomics and proteomics analysis (Weiss T, Taschner S et al., Glia, 2016, PMID: 27545331, DOI: 10.1002/glia.23045; Rifatbegovic F. et al., Int J Canc, 2018, PMID: 28921546, PMCID: PMC5725737, DOI: 10.1002/ijc.31053). Due to donor variation, tumor heterogeneity and higher variability in RNA expression, for RNA-sequencing experiments the following sample sizes were chosen: neuroblastoma tumors without MYCN amplification n=5, tumors with MYCN amplification n=10 , ganglioneuroma Schwann cell stroma-rich areas n=6, cultivated Schwann cells n=5, injured nerve explants n=3. For proteomics analysis, variation within each group was small, as expected, and therefore the following sample sizes were chosen: Ganglioneuroma Schwann cell stroma-rich areas (n=6), neuroblastoma tumors (n=3) neuroblastoma low-passage cell lines STA-NB-7, STA-NB-2 and STA-NB-10 (n=3).<br>For all experiments involving primary cells or tissues or cell lines a sample size of at least n=3 was considered sufficient as this sample size is expected to allow to determine biologically robust changes in cellular states (differentiation, proliferation, apoptosis). Due to donor-dependent variation, in some instances 3 biologically independent replicates, though showing the same result in each replicate, did not result in statistically significant results. In these cases we performed additional experiments.
Data exclusions	Data were excluded only in case of technical failures, i.e. insufficient quality of primary tissue, insufficient cell viability, acquisition/analysis failed, insufficient QC metrics (RNA-sequencing).
Replication	All experiments were performed in independent biological replicates (n≥3). We confirm that all attempts at replication were successful.
Randomization	na - no randomization was performed as this was not a clinical case/control study, but a biomedical basic research study. Therefore, covariates were not considered.
Blinding	For all human samples all patient-related information were removed and samples were pseudonymized. For experimental staining, imaging, FACS and Western blotting analysis, data analysis, transcriptomics and proteomics analysis, only pseudonymized sample codes were used for

researchers performing the wetlab steps and bioinformatics.

Reporting for specific materials, systems and methods

We require information from authors about some types of materials, experimental systems and methods used in many studies. Here, indicate whether each material, system or method listed is relevant to your study. If you are not sure if a list item applies to your research, read the appropriate section before selecting a response.

Materials & experimental systems

n/a	Involved in the study
☐	☒ Antibodies
☐	☒ Eukaryotic cell lines
☒	☐ Palaeontology
☒	☐ Animals and other organisms
☐	☒ Human research participants
☒	☐ Clinical data

Methods

n/a	Involved in the study
☒	☐ ChIP-seq
☐	☒ Flow cytometry
☒	☐ MRI-based neuroimaging

Antibodies

Antibodies used

All antibodies used in this study are listed in supplementary table 3:

1st antibodies Application immunofluorescence flow cytometry Western Blot
Antigen Species catalog No company Clone name dilution comment dilution comment dilution comment
S100B rabbit #Z0311 DAKO polyclonal 1:200 1 hr, RT, perm
Ki67 mouse NCL-Ki67-MM1 Leica Microsystems MM1 1:50 1 hr, RT, perm
Sox10 mouse Sc-365692 Santa Cruz A-2 1:50 o.n., 4°C, perm
vimentin mouse #M0725 DAKO V9   1:200 20 min, 4°C, perm
vimentin chicken #AB5733 Merck Millipore polyclonal 1:200 1 hr, RT, perm
NF200 mouse MAB5266 Merck Milipore N52 1:200 1 hr, RT, perm
NGFR rabbit #8238S CellSignaling D4B3 1:300 o.n., 4°C
S100B-FITC* rabbit #Z0311 DAKO polyclonal   1:50 20 min, 4°C, perm
GD2-FITC* humanized chinese hamster ch14:18 POLYMUN GmbH ch14:18 1:70 o.n., 4°C
GD2-A546* humanized chinese hamster ch14:18 POLYMUN GmbH ch14:18 1:70 o.n., 4°C 1:1000 20 min, 4°C
NF200-A647* mouse MAB5266 Merck Milipore N52   1:400 20 min, 4°C, perm
EGFL8 rabbit #PA5-63929 Thermo Scientific polyclonal 1:100 o.n., 4°C, perm   1:333 o.n., 4°C
CD3 mouse #C7048 Sigma-Aldrich UCHT-1 1:50 o.n., 4°C
HLA-DR-α1 mouse #M0746 DAKO TAL-1B5 1:50 o.n., 4°C
c-JUN rabbit #9165 CellSignaling 60A8 1:100 o.n., 4°C, perm
GAPDH mouse #32233 Santa Cruz 6C5   1:2000 1h, RT

2nd antibodies Application immunofluorescence flow cytometry Western Blot
Antigen Species catalog No company dilution comment dilution comment dilution comment
α rb FITC swine #F0205 DAKO polyclonal 1:50 1 hr, RT
α ms AF594 goat #A11032 LifeTech. polyclonal 1:300 1 hr, RT 1:1000 20 min, 4°C
α ch AF647 goat #SA5-10073 LifeTech. polyclonal 1:300 1 hr, RT
α ms IRdye680LT goat P/N 925-68020 LI-COR polyclonal 1:10000 1h, RT
α rb HRP goat #7074 Cell Signaling polyclonal 1:1000 10 min, RT

Click-iT EdU Alexa Fluor 647 Flow Cytometry Assay Kit Thermo Fisher Scientific #C10419

Validation

All antibodies used were previously tested by the manufacturers and confirmed to specifically recognize the protein as indicated on their web site, product sheets and by images provided.

S100B #Z0311 DAKO
Reacts strongly with human S100B, and weakly or very weakly with S100A1 and S100A6, respectively. S100 from ox brain has been used for the immunization. Z0311 labels glial cells in the brain and ependymal cells. Moreover, Schwann's cells of the peripheral nervous system are positive. Results aid in the classification of tumors in central and peripheral nervous system, such as schwannomas, ependydomas as well as in different grades of astrogliomas, also including glioblastomas. A large proportion of cells in human tumors originating from different salivary glands are labeled by anti-S100. The antibody is also a useful aid for classification of malignant melanocytic tumors of the skin and metastases of human malignant melanomasm https://www.agilent.com/en/product/immunohistochemistry/antibodies-controls/primary-antibodies/s100-(dako-omnis)-76198

Ki67 NCL-Ki67-MM1 Leica Microsystems
Normal Tissues Clone MM1 detected the Ki67 antigen in the nucleus of proliferating cells in 11/44 normal cases evaluated,

including proliferating cells within crypts of ileum, cecum, colon and rectum, proliferating cells within lymph node, thymus, para and suprabasal cells of the tongue, epithelial and stromal cell elements of pro-endometrium, germinal center elements of tonsil, ductal cell elements of breast and occasional cells in supra and parabasal layers of esophagus. (Total number of normal cases evaluated = 44). Abnormal Tissues Clone MM1 stained 26/70 tumors evaluated, including breast tumors (12/29, including 11/26 ductal carcinomas and 1/1 medullary carcinoma), lung tumors (3/4, including 2/2 non-small cell carcinoma and 1/1 squamous cell carcinoma), ovarian tumors (2/4, including 2/2 cystadenocarcinomas), thyroid papillary carcinomas (1/4), liver tumors (1/4, including 1/1 metastatic carcinomas), squamous cell carcinomas of the cervix (2/2), colon adenocarcinomas (2/2), renal cell carcinomas (1/2), squamous cell carcinomas of the esophagus (1/1), squamous cell carcinomas of the tongue (1/2), brain tumors (0/2), adenocarcinomas of the stomach (0/2), soft tissue tumors (0/2), metastatic tumors of unknown origin (0/2), testicular seminomas (0/2), adenocarcinomas the rectum (0/2), skin tumors (0/2), a squamous cell carcinoma of the larynx (0/1), an atypical carcinoid tumor of the thymus (0/1). (Total number of tumor cases evaluated = 70). NCL-L-Ki67-MM1 is recommended for the assessment of cell proliferation in normal and neoplastic tissues.

file:///C:/Users/sabine.taschner/Downloads/ki67-mm1-l-ce.pdf

Sox10 Sc-365692 Santa Cruz

Sox-10 (A-2): sc-365692. Immunoperoxidase staining of formalin fixed, paraffin-embedded human prostate tissue showing nuclear staining of glandular cells.

Sox-10 (A-2) is recommended for detection of Sox-10 of mouse, rat and human origin by Western Blotting (starting dilution 1:100, dilution range 1:100-1:1000), immunoprecipitation [1-2 µg per 100-500 µg of total protein (1 ml of cell lysate)], immunofluorescence (starting dilution 1:50, dilution range 1:50-1:500), immunohistochemistry (including paraffin-embedded sections) (starting dilution 1:50, dilution range 1:50-1:500) and solid phase ELISA (starting dilution 1:30, dilution range 1:30-1:3000). Sox-10 (A-2) is also recommended for detection of Sox-10 in additional species, including canine, bovine and porcine. Molecular Weight of Sox-10: 58 kDa. Positive Controls: SK-MEL-24 whole cell lysate: sc-364259, C6 whole cell lysate: sc-364373 or A-375 cell lysate: sc-3811.

DATA SELECT PRODUCT CITATIONS 1. Keith, J.L., et al. 2013. Clinical neuropathology practice guide 6-2013: morphology and an appropriate immunohistochemical screening panel aid in the identification of synovial sarcoma by neuropathologists. Clin. Neuropathol. 32: 461-470. 2. Patowary, A., et al. 2019. Family-based exome sequencing and case-control analysis implicate CEP41 as an ASD gene. Transl. Psychiatry 9: 4. 3. Planells, B., et al. 2019. Gene expression profiles of bovine genital ridges during sex determination and early differentiation of the gonads. Biol. Reprod. E-published.

Images: Sox-10 (A-2): sc-365692. Immunofluorescence staining of formalin-fixed Hep G2 cells showing nuclear localization (A). Immunoperoxidase staining of formalin fixed, paraffin-embedded human salivary gland tissue showing nuclear and cytoplasmic staining of glandular cells (B).

https://datasheets.scbt.com/sc-365692.pdf

Vimentin #M0725 DAKO

Specificity: In Western blotting of purified porcine vimentin, the antibody labels a single band of 57 kDa corresponding to vimentin. When applying whole cell extracts of cell lines expressing vimentin plus glial fibrillary acidic protein (GFAP), and vimentin plus desmin, respectively, the antibody labels specifically the 57 kDa vimentin band. As directly shown by these experiments, the antibody does not react with the two IF proteins most closely related to vimentin, i.e. desmin and GFAP (3). In IHC, the antibody labels the vimentin-positive human cell lines IMR90, RD, glioma and HeLa (3). See package insert for reference (s).

References 1. Kiernan JA. Histological and Histochemical Methods: Theory and Practice. New York: Pergamon Press 1981 2. Sheehan DC and Hrapchak BB. Theory and Practice of Histotechnology. St. Louis: C.V. Mosby Co. 1980 3. Diamandis EP, Schwartz MK. Tumor Markers: Physiology, Pathobiology, Technology, and Clinical Applications. Washington DC: AACC Press 2002 4. Dabbs DJ. Diagnostic Immunohistochemistry. Philadelphia: Churchill Livingstone Elsevier 2002 5. Clinical Laboratory Improvement Amendments of 1988: Final Rule, 57 CFR 7163, February 28, 1992 6. Key M (ed.). Education Guide: Immunohistochemical Staining Methods, Fourth Edition. Carpinteria: Dako 2006 7. Nadji M and Morales AR. Immunoperoxidase. Part I: The technique and its pitfalls. Lab Med 1983; 14:767 8. Banks PM. Diagnostic applications of an immunoperoxidase method in hematopathology. J Histochem Cytochem 1979; 27:1192-94 9. Culling CFA, Reid PE, Sinnott NM. The effect of various fixatives and trypsin digestion upon the staining of routine paraffin-embedded sections by the peroxidase-antiperoxidase and immunofluorescent technique. J Histotech 1980; 3:10-19 10. Carson FL (ed.). Histotechnology: A self-instructional text. Chicago: ASCP Press 1990; 22 11. Grabau DA, Nielsen O, Hansen S, Nielsen MM, Lænkholm A-V, Knoop A, Pfeiffer P. Influence of storage temperature and hightemperature antigen retrieval buffers on results of immunohistochemical staining in sections stored for long periods. Appl Immunohistochem 1998; 6(4):209-13 12. Shi SR, Key ME, Kalra KL. Antigen retrieval in formalin-fixed, paraffin-embedded tissues: An enhancement method for immunohistochemical staining based on microwave oven heating of tissue sections. J Histochem Cytochem 1991; 39:741-48 13. Koopal SA, Coma MI, Tibosch AMG, Surmeijer AJH. Low temperature heating overnight in Tris-HCl buffer pH 9 is a good alternative for antigen retrieval in formalin-fixed paraffin-embedded tissue. Appl Immunohistochem 1998; 6:228-33 14. National Committee for Clinical Laboratory Standards. Protection of laboratory workers from instrument biohazards and infectious disease transmitted by blood, body fluids, and tissue; approved guideline. Villanova, PA 1997: Order code M29-A 15. National Committee for Clinical Laboratory Standards. Quality assurance for immunocytochemistry; approved guideline. Villanova, PA, 1999; 19(26):Order code MM4-A 16. Koretz K, Lemain ET, Brandt I, and Moller P. Metachromasia of 3-amino-9-ethylcarbazole (AEC) and its prevention in immunoperoxidase techniques. Histochem 1987; 86:471-78 17. Elias JM, Gown AM, Nakamura RM, Wilbur DC, Herman GE, Jaffe ES, Battifora H, Brigati DJ. Special report: Quality control in immunohistochemistry. Amer J Clin Pathol 1989; 92:836-43 18. Omata M, Liew CT, Ashcavai M, Peters RL. Nonimmunologic binding of horseradish peroxidase to hepatitis B surface antigen: a possible source of error in immunohistochemistry. Amer J Clin Pathol 1980; 73:626-32 19. Herman GE and Elfont EA. The taming of immunohistochemistry: The new era of quality control. Biotech Histochem 1991; 66:194-99

(https://www.agilent.com/cs/library/packageinsert/public/108007002.PDF)

Vimentin #AB5733 Merck Millipore

REFERENCE: Endotoxin-induced inflammation down-regulates L-type amino acid transporter 1 (LAT1) expression at the blood-brain barrier of male rats and mice.

Wittmann, G; Mohácsik, P; Balkhi, MY; Gereben, B; Lechan, RM
Fluids and barriers of the CNS 12 21 2015
Semaphorin7A regulates neuroglial plasticity in the adult hypothalamic median eminence.
Parkash, J; Messina, A; Langlet, F; Cimino, I; Loyens, A; Mazur, D; Gallet, S; Balland, E; Malone, SA; Pralong, F; Cagnoni, G; Schellino, R; De Marchis, S; Mazzone, M; Pasterkamp, RJ; Tamagnone, L; Prevot, V; Giacobini, P
Nature communications 6 6385 2015
Zebrafish adult-derived hypothalamic neurospheres generate gonadotropin-releasing hormone (GnRH) neurons.
Cortés-Campos, C; Letelier, J; Ceriani, R; Whitlock, KE
Biology open 4 1077-86 2015
Inhibition of TGFβ signaling increases direct conversion of fibroblasts to induced cardiomyocytes.
Ifkovits, JL; Addis, RC; Epstein, JA; Gearhart, JD
PloS one 9 e89678 2014
Spatially restricted Hedgehog signalling regulates HGF-induced branching of the adult prostate.
Lim, A; Shin, K; Zhao, C; Kawano, S; Beachy, PA
Nature cell biology 16 1135-45 2014
Dicer is required for maintenance of adult pancreatic acinar cell identity and plays a role in Kras-driven pancreatic neoplasia.
Wang, YJ; McAllister, F; Bailey, JM; Scott, SG; Hendley, AM; Leach, SD; Ghosh, B
PloS one 9 e113127 2014
Collagen signaling enhances tumor progression after anti-VEGF therapy in a murine model of pancreatic ductal adenocarcinoma.
Aguilera, KY; Rivera, LB; Hur, H; Carbon, JG; Toombs, JE; Goldstein, CD; Dellinger, MT; Castrillon, DH; Brekken, RA
Cancer research 74 1032-44 2014
A mammary stem cell population identified and characterized in late embryogenesis reveals similarities to human breast cancer.
Spike, BT; Engle, DD; Lin, JC; Cheung, SK; La, J; Wahl, GM
Cell stem cell 10 183-97 2012
GFAPδ expression in glia of the developmental and adolescent mouse brain.
Mamber, C; Kamphuis, W; Haring, NL; Peprah, N; Middeldorp, J; Hol, EM
PloS one 7 e52659 2012
The Expression Pattern of the Na(+) Sensor, Na(X) in the Hydromineral Homeostatic Network: A Comparative Study between the Rat and Mouse.
Nehmé, B; Henry, M; Mouginot, D; Drolet, G
Frontiers in neuroanatomy 6 26 2012
Repression of a potassium channel by nuclear hormone receptor and TGF-β signaling modulates insulin signaling in Caenorhabditis elegans.
Park, D; Jones, KL; Lee, H; Snutch, TP; Taubert, S; Riddle, DL
PLoS genetics 8 e1002519 2012
Dementia in Parkinson's Disease Correlates with α-Synuclein Pathology but Not with Cortical Astrogliosis.
van den Berge, SA; Kevenaar, JT; Sluijs, JA; Hol, EM
Parkinson's disease 2012 420957 2012
GFAP isoforms in adult mouse brain with a focus on neurogenic astrocytes and reactive astrogliosis in mouse models of Alzheimer disease.
Kamphuis, W; Mamber, C; Moeton, M; Kooijman, L; Sluijs, JA; Jansen, AH; Verveer, M; de Groot, LR; Smith, VD; Rangarajan, S; Rodríguez, JJ; Orre, M; Hol, EM
PloS one 7 e42823 2012
Inducible expression of TGFβ, Snail and Zeb1 recapitulates EMT in vitro and in vivo in a NSCLC model.
Argast GM, Krueger JS, Thomson S, Sujka-Kwok I, Carey K, Silva S, O'Connor M, Mercado P, Mulford IJ, Young GD, Sennello R, Wild R, Pachter JA, Kan JL, Haley J, Rosenfeld-Franklin M, Epstein DM.
Clinical & experimental metastasis 28 593-614 2011
Cooperative signaling between oncostatin M, hepatocyte growth factor and transforming growth factor-β enhances epithelial to mesenchymal transition in lung and pancreatic tumor models.
Gretchen M Argast,Peter Mercado,Iain J Mulford,Matthew O'Connor,David M Keane,Salam Shaaban,David M Epstein,Jonathan A Pachter,Julie L C Kan
Cells, tissues, organs 193 2011
A systems view of epithelial-mesenchymal transition signaling states.
Thomson, S; Petti, F; Sujka-Kwok, I; Mercado, P; Bean, J; Monaghan, M; Seymour, SL; Argast, GM; Epstein, DM; Haley, JD
Clinical & experimental metastasis 28 137-55 2011
p53-dependent regulation of growth, epithelial-mesenchymal transition and stemness in normal pancreatic epithelial cells.
Andreia V Pinho,Ilse Rooman,Francisco X Real
Cell cycle (Georgetown, Tex.) 10 2011
Gap junction adhesion is necessary for radial migration in the neocortex.
Elias, Laura A B, et al.
Nature, 448: 901-7 (2007) 2007
(https://www.merckmillipore.com/AT/de/product/Anti-Vimentin-Antibody,MM_NF-AB5733#anchor_COA)

NF200 MAB5266 Merck Milipore
Specificity: MAB5266 reacts with the phophorylated and dephosphorylated H-chain of neurofilament 200kDa (NF-H) in normal tissues/extracts (Shaw, 1986). MAB5266 can be used to detect cells of neuronal origin by immunohistochemistry and Western

blot. It has been reported that reactivity of MAB5266 with NF-H is blocked in cdk-5 over-expressing cells (Guidato, 1996).
Application Notes: Immunohistochemistry: 5-10 µg/mL
Immunohistochemistry: Antibody N52 reacts with a fragment of NF-200 side-arm that is located at the end of the MPR KSP domain and which contains the consensus cdk-5 phosphorylation site, however this reactivity is abolished if the NF-200 fragment becomes phosphorylated by cdk-5 {Guidato et al, 1996}. Thus for the fullest staining and reactivity, including westerns, it is suggested that samples be treated with alkaline phosphatase prior to antibody staining.
Literatur
Selective conversion of fibroblasts into peripheral sensory neurons.
Blanchard, JW; Eade, KT; Szűcs, A; Lo Sardo, V; Tsunemoto, RK; Williams, D; Sanna, PP; Baldwin, KK
Nature neuroscience 18 25-35 2015
Neurotrophin-4 regulates the survival of gustatory neurons earlier in development using a different mechanism than brain-derived neurotrophic factor.
Patel, AV; Krimm, RF
Developmental biology 365 50-60 2012
Sensory-motor deficits and neurofilament disorganization in gigaxonin-null mice.
Ganay, T; Boizot, A; Burrer, R; Chauvin, JP; Bomont, P
Molecular neurodegeneration 6 25 2011
The BTB and CNC homology 1 (BACH1) target genes are involved in the oxidative stress response and in the control of the cell cycle
Warnatz HJ, Schmidt D, Manke T, Piccini I, Sultan M, Borodina T, Balzereit D, Wruck W, Soldatov A, Vingron M, Lehrach H, Yaspo ML
J Biol Chem 2011
BDNF is required for the survival of differentiated geniculate ganglion neurons.
Patel, Ami V and Krimm, Robin F
Dev. Biol., 340: 419-29 (2010) 2010
Contribution of voltage-gated sodium channels to the b-wave of the mammalian flash electroretinogram.
Mojumder, DK; Sherry, DM; Frishman, LJ
The Journal of physiology 586 2551-80 2008
Subcellular compartmentalization of two calcium binding proteins, calretinin and calbindin-28 kDa, in ganglion and amacrine cells of the rat retina.
Mojumder, DK; Wensel, TG; Frishman, LJ
Molecular vision 14 1600-13 2008
Aminoglycoside-induced degeneration of adult spiral ganglion neurons involves differential modulation of tyrosine kinase B and p75 neurotrophin receptor signaling.
Justin Tan, Robert K Shepherd, Justin Tan, Robert K Shepherd, Justin Tan, Robert K Shepherd, Justin Tan, Robert K Shepherd
The American journal of pathology 169 528-43 2006
Effects of spinal nerve ligation on immunohistochemically identified neurons in the L4 and L5 dorsal root ganglia of the rat.
Hammond, Donna L, et al.
J. Comp. Neurol., 475: 575-89 (2004) 2004
Adeno-associated viral transfer of opioid receptor gene to primary sensory neurons: a strategy to increase opioid antinociception.
Xu, Y, et al.
Proc. Natl. Acad. Sci. U.S.A., 100: 6204-9 (2003) 2003
https://www.merckmillipore.com/AT/de/product/Anti-Neurofilament-200-kDa-Antibody-clone-N52,MM_NF-MAB5266#anchor_REF

NGFR #8238S CellSignaling
Image: Confocal immunofluorescent analysis of SK-N-MC cells using p75NTR (D4B3) XP® Rabbit mAb (green). Actin filaments were labeled with DY-554 phalloidin (red). Blue pseudocolor = DRAQ5® #4084 (fluorescent DNA dye).
Specificity / Sensitivity
p75NTR (D4B3) XP® Rabbit mAb recognizes endogenous levels of total p75NTR protein.
Species Reactivity: Human, Mouse, Rat
Journal:Biol Open
Applications:Immunocytochemistry (ICC)
Reactivity:Homo sapiens (Human)
Title:Feeder-free differentiation of cells exhibiting characteristics of corneal endothelium from human induced pluripotent st...
Author:Michael D Wagoner, et. al.
Year:2018
Journal:Mol Syst Biol
Applications:Immunofluorescence (IF)
Reactivity:Homo sapiens (Human)
Title:Adaptive resistance of melanoma cells to RAF inhibition via reversible induction of a slowly dividing de-differentiated ...
Author:Mohammad Fallahi-Sichani, et. al.
Year:2017
https://www.cellsignal.com/products/primary-antibodies/p75ntr-d4b3-xp-rabbit-mab/8238

GD2 ch14:18 POLYMUN GmbH
Literature: MAbs. 2013 Sep 1; 5(5): 801–809.
Published online 2013 May 31. doi: 10.4161/mabs.25215
PMCID: PMC3851232
PMID: 23924804
Ch14.18 antibody produced in CHO cells in relapsed or refractory Stage 4 neuroblastoma patients
A SIOPEN Phase 1 study
Ruth Ladenstein, 1 ,* Silke Weixler, 2 Bianca Baykan, 3 Matthias Bleeke, 3 Renate Kunert, 4 Dietmar Katinger, 5 Ingrid Pribill, 1 Petra Glander, 6 Steffen Bauer, 6 Vito Pistoia, 7 Jean Michon, 8 Alberto Garaventa, 9 and Holger N. Lode 3

EGFL8 #PA5-63929 Thermo Scientific
Image: Immunofluorescent staining of EGFL8 in human cell line U-2 OS using EGFL8 Polyclonal Antibody (Product # PA5-63929) shows localization to vesicles.
Immunohistochemical staining of EGFL8 in human placenta shows strong membranous and cytoplasmic positivity in decidual cells. Samples were probed using an EGFL8 Polyclonal Antibody (Product # PA5-63929).
Specificity: human
Immunogen sequence: FNTAGSFTCG CPHDLVLGVD GRTCMEGSPE PPTSASILSV AVREAEKDER ALKQEIHELR G

Highest antigen sequence identity to the following orthologs: Mouse - 71%, Rat - 66%.
Applications
Tested Dilution
Immunocytochemistry (ICC) 0.25-2 µg/mL
Immunofluorescence (IF) 0.25-2 µg/mL
Immunohistochemistry (IHC) 1:50-1:200
https://www.thermofisher.com/antibody/product/EGFL8-Antibody-Polyclonal/PA5-63929

CD3 #C7048 Sigma-Aldrich
Monoclonal Anti-Human CD3 recognizes the CD3 complex which is composed of 5 chains designated g, d, e, z and h having a molecular mass distribution of 16, 20, and 25 - 28 kDa. The CD3 human lymphocyte surface antigen is a glycoprotein thought to be associated with the T cell antigen receptor and to be involved in transmission of activation signals. The CD3 antigen is present on 60-80% of normal peripheral blood mononuclear cells, 20-40% of normal spleen cells, 40% of normal thymocytes, the majority of T-CLL, and approximately 70% of T-ALL. It is detectable in the cytoplasm of cortical thymocytes but also appears on the surface of medullary thymocytes. The antibody stains the cytoplasm of cerebellar Purkinje cells but does not stain B lymphocytes, monocytes, granulocytes or NK cells. The epitope recognized by clone UCHT-1 is expressed on the e-chain of the CD3 antigen/T cell receptor complex. Detection of the epitope appears to be dependent of the binding to CD3-g or CD3-d. The epitope is sensitive to routine formalin fixation and paraffin embedding. Cryostat sections post fixed in formalin can be stained.
References Beverley, P. C., and Callard, R. E., Eur. J. Immunol., 11, 329 (1981). Leucocyte Typing IV, Knapp, W., et al., (eds.), Oxford Press, New York, 1075 (1989). Callard, R. E., et al., Clin. Exp. Immunol., 43, 497 (1981). Garson, J. A., et al., Nature, 298, 375 (1982). Burns, G. F., et al., J. Immunol., 129, 1451 (1982). Reinherz, E. L., et al., Cell, 30, 735 (1982). Borst, J., et al., J. Biol. Chem., 258, 5135 (1983). Meuer, S. C., et al., Nature, 303, 808 (1983). van dan Elsen, P., et al., Nature, 312, 413 (1984). Furley, A., et al., Cell, 46, 75 (1986). Gold, D. P., et al., Nature, 321, 431 (1986). Oettgen, H., et al., Annu. Rev. Immunol., 6, 629 (1988)
https://www.sigmaaldrich.com/content/dam/sigma-aldrich/docs/Sigma/Datasheet/3/c7048dat.pdf

HLA-DR-α1 #M0746 DAKO
Immunogen 33 kDa-chain subunit of HLA-D products prepared from the B lymphoblastoid cell line Bristol 8 (2).
Specificity: The antibody reacts with the monomorphic-chain of HLA Class II DR antigens. The epitope is localized in the C-terminal intracellular tail of DR-chain (3). HLA-D antigens defined by this antibody are present on a wide variety of normal cells of different histological types. The antibody reacts with B cells, activated T cells, macrophages, antigen presenting cells, e.g. Langerhans' cells of skin as well as some endothelial and epithelial tissues (7). Cross-reactivity to porcine lymphoid tissues (4) and to canine lymphocytes (5) has been described.
Performance characteristics Cells labelled by the antibody display strong of the cell surface membrane, but also intracellular staining is observed (8).
References

1. Navarrete CV. The HLA system in blood transfusion. Baillière's Clinical Haematology 2000;13,511-32.
2. Adams TE, Bodmer JG, Bodmer WF. Production and characterization of monoclonal antibodies recognizing the alpha-chain subunits of human Ia alloantigens. Immunology 1983;50:613-24.
3. Grüneberg U, Rich T, Roucard C, Marieke van Ham S, Charron D, Trowsdale J. Two widely used antiDR monoclonal antibodies bind to an intracellular C-terminal epitope. Hum Immunol 1997;53:34-8.
4. Tanimoto T, Ohtsuki Y. Evaluation of antibodies reactive with porcine lymphocytes and lymphoma cells in formalin-fixed, paraffin-embedded, antigen-retrieved tissue sections. Am J Vet Res 1996;57:853-9.
5. Galkowska H, Waldemar LO, Wojewodzka U. Reactivity of antibodies directed against human antigens with surface markers on canine leukocytes. Vet Immunol Immunopathol 1996;53:329-34.
6. Barclay AN, Brown MH, Law SKA, McKnight AJ, Tomlinson MG and van der Merve PA in: The Leucocyte
Antigen Factsbook. MHC Class II. Hartcourt Brace & Company, Publishers. 1997:567-8.
7. Thomas A, Lindsay J, Wilkinson M and Bodmer J. HLA-D region–chain monoclonal antibodies: Crossreaction between an anti-DP–chain antibody and smooth muscle. J Pathol 1988;154:353-63.
8. Norton AJ, Isaacson PG. Detailed phenotypic analysis of B-cell lymphoma using a panel of antibodies reactive in routinely fixed wax-embedded tissue. Am J Pathol 1987;128,225-40.

https://www.agilent.com/cs/library/packageinsert/public/SSM0746RUO-E_01.pdf

c-JUN
images: Confocal immunofluorescent analysis of HeLa cells, using c-Jun (60A8) Rabbit mAb (green). Actin filaments have been labeled with Alexa Fluor® 555 phalloidin (red).
REACTIVITY H M R Mk
Application Dilution
Immunofluorescence (Immunocytochemistry) 1:400
Specificity / Sensitivityc-Jun (60A8) Rabbit mAb detects endogenous levels of total c-Jun protein, regardless of phosphorylation state.
Species Reactivity:Human, Mouse, Rat, Monkey
References:
Journal:J Cell Biol
Applications:Immunofluorescence (IF)
Reactivity:Homo sapiens (Human)
Title:Cadherin complexes recruit mRNAs and RISC to regulate epithelial cell signaling.
Author:Antonis Kourtidis, et. al.
Year:2017
Journal:Mol Syst Biol
Applications:Immunofluorescence (IF)
Reactivity:Homo sapiens (Human)
Title:Adaptive resistance of melanoma cells to RAF inhibition via reversible induction of a slowly dividing de-differentiated ...
Author:Mohammad Fallahi-Sichani, et. al.
Year:2017
Journal:Diabetes
Applications:Immunofluorescence (IF), Immunohistochemistry...
Reactivity:Homo sapiens (Human)
Title:High Glucose Stimulates Tumorigenesis in Hepatocellular Carcinoma Cells Through AGER-Dependent O-GlcNAcylation of c-Jun.
Author:Yongxia Qiao, et. al.
Year:2016
Journal:Mol Syst Biol
Applications:Immunofluorescence (IF)
Reactivity:Homo sapiens (Human)
Title:Systematic analysis of BRAF(V600E) melanomas reveals a role for JNK/c-Jun pathway in adaptive resistance to drug-induced...
Author:Mohammad Fallahi-Sichani, et. al.
Year:2015
https://www.cellsignal.com/products/primary-antibodies/c-jun-60a8-rabbit-mab/9165

GAPDH #32233 Santa Cruz
Image: GAPDH (6C5): sc-32233.Immunofluorescence staining of methanol-fixed HeLa cells showing cytoplasmic localization.
SOURCE
GAPDH (6C5) is a mouse monoclonal antibody raised against GAPDH purified from muscle of rabbit origin.
APPLICATIONS
GAPDH (6C5) is recommended for detection of GAPDH of mouse, rat, human, rabbit and Xenopus laevis origin by Western Blotting (starting dilution 1:200, dilution range 1:100-1:1000), immunoprecipitation [1-2 µg per 100-500 µg of total protein (1 ml of cell lysate)] and immunofluorescence (starting dilution 1:50, dilution range 1:50-1:500).
Molecular Weight of GAPDH: 37 kDa.
SELECT PRODUCT CITATIONS
1. Chang, M.C., et al. 2004. The induction of prostaglandin E2 production, interleukin-6 production, cell cycle arrest, and cytotoxicity in primary oral keratinocytes and KB cancer cells by areca nut ingredients is differentially regulated by MEK/ERK activation. J. Biol. Chem. 279: 50676-50683.
2. Shyu, K.G., et al. 2004. Saikosaponin C induces endothelial cells growth, migration and capillary tube formation. Life Sci. 76: 813-826.
3. Lin, S., et al. 2004. Berberine inhibits HIF-1a expression via enhanced proteolysis. Mol. Pharmacol. 66: 612-619.
4. Martincuks, A., et al. 2017. Nuclear translocation of STAT3 and NFkB are independent of each other but NFkB supports expression and activation of STAT3. Cell. Signal. 32: 36-47.
5. Arai, S., et al. 2017. Functional loss of DHRS7C induces intracellular Ca2+ overload and myotube enlargement in C2C12 cells via calpain activation. Am. J. Physiol., Cell Physiol. 312: C29-C39.

6. Tahrir, F.G., et al. 2017. Evidence for the role of BAG3 in mitochondrial quality control in cardiomyocytes. J. Cell. Physiol. 232: 797-805.
7. Ferguson, K.T., et al. 2017. The novel mTOR complex 1/2 inhibitor P529 inhibits human lung myofibroblast differentiation. J. Cell. Biochem. E-published.
8. Márton, M., et al. 2017. A systems biological view of life-and-death decision with respect to endoplasmic reticulum stress-the role of PERK pathway. Int. J. Mol. Sci. E-published.
9. Abdelmohsen, K., et al. 2017. Identification of HuR target circular RNAs uncovers suppression of PABPN1 translation by CircPABPN1. RNA Biol. E-published.
https://datasheets.scbt.com/sc-32233.pdf

In addition, we performed the following control experiments for each primary antibody: negative control: secondary antibody only or isotype control to estimate background staining. Weak and difuse background staining was observed for all tested primary antibodies. Positive control: tissue or cell type reported to express the particular protein or recombinant protein (in case of EGFL8 Western blotting). Each antibody was tested in single staining experiments before applied in combination.
Positive controls:
S100B human cultured Schwann cells
Ki67 human cultured neuroblastoma cell line STA-NB-6 and CLB-Ma
Sox10 injured nerve tissue
Vimentin human cultured nerve associated fibroblasts
Vimentin human cultured nerve associated fibroblasts
NF200 human cultured neuroblastoma cell line STA-NB-6 and CLB-Ma
S100B-FITC human cultured Schwann cells
GD2-FITC human cultured neuroblastoma cell line STA-NB-6 and CLB-Ma
GD2-A546 human cultured neuroblastoma cell line STA-NB-6 and CLB-Ma
NF200-A647 human cultured neuroblastoma cell line STA-NB-6 and CLB-Ma
NGFR human cultured Schwann cells
EGFL8 human cultivated neuroblastoma cell lines STA-NB-6 and CLB-MA cell lysate and culture supernatants
CD3 T-lymphocytes infiltrating neuroblastoma tumor tissue
HLA-DR-α1 myeloid cells infiltrating neuroblastoma tumor tissue
c-JUN injured nerve tissue
GAPDH human cultured neuroblastoma cell line STA-NB-6 and CLB-Ma

Eukaryotic cell lines

Policy information about cell lines

Cell line source(s)

STA-NB-2, STA-NB-6, STA-NB-7, STA-NB-10, STA-NB-15: in house patient-derived cell lines.
CLB-Ma: provided by V. Combaret (establisher of the cell line).
IMR5 were: provided by J. Khan, NIH, US.
SH-SY5Y: provided by J.L. Biedler and B.A. Spengler, Memorial Sloan Kettering Cancer Center, US (establisher of the cell line)

Authentication

Cells line authentication was done along genetic characterization by high-density SNP array (Affymetrix).

Mycoplasma contamination

We confirm that all cell lines have been tested negative for Mycoplasma contamination.

Commonly misidentified lines
(See ICLAC register)

No commonly misientified line has been used in this study.

Human research participants

Policy information about studies involving human research participants

Population characteristics	This is an exploratory, basic biomedical research study. Information on covariates is available (age, gender, genetic characteristics), but are currently not fully reported in the manuscript. Human peripheral nerve explants were collected from adolescents and adults (age please see below). Tumor specimen were derived from children, adolescents and young adults as neuroblastic tumors only occur in this age group. Biosamples from both, male and female, patients were included. Due to anonymization, previous diseases are not known. Neuroblastoma tumor specimen were selected to represent major genetic subtypes, i.e. cases with and without MYCN amplification. Tumor specimen from male and female patients between 0 and 18 years of age diagnosed with neuroblatoma (NB-Tumor, n=15) and ganglioneuroma (GN-Tumor, n=6) have been collected during surgery or biopsy for diagnostic purposes and left-overs were cryopreserved and were accessible through the Children's Cancer Research Institute Biobank (Vienna, Austria) for analysis.<br>Human peripheral nerves were collected during reconstructive surgery, amputations or organ donations of male and female patients between 16 and 70 years of age.
Recruitment	Patients were recruited based on the inclusion criteria listed above. Neuroblastoma and ganglioneuroma samples were selected based on availabilility of biobanked material. Thus, a selection bias might be the availability of sufficient biopsy material and thereby exclusion of patients where surgery was not possible, e.g. due to critical health condition or inoperable tumor, is possible.
Ethics oversight	The collection and research use of human peripheral nerve tissues and human tumor specimen was conducted according to the guidelines of the Council for International Organizations of Medical Sciences (CIOMS) and World Health Organisation (WHO) and has been approved by the local ethics committees of the Medical University of Vienna (EK2281/2016 and 1216/2018). Written informed consent has been obtained from parents/guardians/legally authorized representatives.

Note that full information on the approval of the study protocol must also be provided in the manuscript.

Flow Cytometry

Plots

Confirm that:

- [x] The axis labels state the marker and fluorochrome used (e.g. CD4-FITC).
- [x] The axis scales are clearly visible. Include numbers along axes only for bottom left plot of group (a 'group' is an analysis of identical markers).
- [x] All plots are contour plots with outliers or pseudocolor plots.
- [x] A numerical value for number of cells or percentage (with statistics) is provided.

Methodology

Sample preparation	All antibody details are listed above and in S.Table 3. If not stated otherwise, all steps of the staining procedures were performed on ice. The following antibodies have been conjugated to fluorochromes using commercially available kits according to the manufacturer´s instructions: anti-S100B has been conjugated to FITC (FLUKA) using Illustra NAP-5 columns (GE Healthcare), anti-GD2 (ch14:18, kindly provided by Professor Rupert Handgretinger, Department of Hematology/Oncology, Children's University Hospital, Tübingen, Germany) has been conjugated to AF546 using the AlexaFluor® 546 protein labeling kit (Molecular probes) and anti-NF200 has been conjugated to AF647 using the AlexaFluor® 647 protein labeling kit (Molecular probes).<br>Cells were detached using Accutase (LifeTechnologies) and washed with FACS-buffer (1x PBS containing 0.1% BSA and 0.05% NaAzide).<br>For the differentiation FACS panel, cells were incubated with GD2-AF546 for 20 min, washed once with FACS-buffer and fixed using Cytofix/Cytoperm (BD Biosciences) in the dark for 20 min. After washing with 1x perm/wash (BD), cells were stained with anti-S100B-FITC and NF200-A647 for 20 min. Cells were washed in 1x perm/wash and analyzed immediately at the FACSFortessa flow cytometer equipped with the FACSDiva software (both BD).<br>For the proliferation FACS panel, 1 µM EdU was added to cultures for about 15 hours. Cells were detached, washed and fixed in Roti-Histofix 4% for 20 min at RT. Permeabilization and EdU detection was carried out using the Click-iT EdU Alexa Fluor 647 Flow Cytometry Assay Kit (Thermo Fisher Scientific) according to the manufacturer's manual. Additional extracellular/intracellular staining was performed with GD2-A546 and anti-S100B-FITC antibodies in 1x saponin-based perm/wash for 30 min. After washing, cells were resuspended in 1x saponin-based perm/wash, 1 µl of FxCycle Violet (LifeTechnologies) DNA dye was added and samples were analyzed immediately at the FACSFortessa.
Instrument	FACSFortessa flow cytometer equipped with 5 lasers (355, 405, 488, 561 and 640 nm) (Becton Dickinson).
Software	FACS acquisition and analysis: FACSDiva software version 8.0 (Becton Dickinson)
Cell population abundance	FACS sorting has not been performed in this study.
Gating strategy	A hierarchical gating strategy ´has been applied as follows:<br>Neuronal differentiation (Figures 4b and c and 6b,c,d,f): 1. FSC/SSC: gating on a defined, clearly seperate from smaller events "intact cell" population. 2. S100-FITC/GD2-A546: gating on a defined, clearly distinct S100positive GD2negative "Schwann cell" and S100negative GD2 positive "neuroblastoma" population. 3. Mean flourescence intensity of NF200-A647 was determined in

the "neuroblastoma" population.
Proliferation (Figures (Figures 5a, b, c and 6b,c,d,f): 1. FSC/SSC: gating on a defined, clearly seperate from smaller events "intact cell" population. 2. S100-FITC/GD2-A546: gating on a defined, clearly distinct S100positive GD2negative "Schwann cell" and S100negative GD2 positive "neuroblastoma" population. 3. FxCycle Violet/EdU-A647: Within the n"neuroblastoma" cells, the distinct EdU positive cell population was gated and presented as percentage of proliferating cells.

☒ Tick this box to confirm that a figure exemplifying the gating strategy is provided in the Supplementary Information.
