# Supplementary material for: Schwann cell plasticity regulates neuroblastic tumor cell differentiation via epidermal growth factor-like protein 8
Source: Nat Commun. 2021 Mar 12;12:1624. doi: 10.1038/s41467-021-21859-0 (PMC7954855; doi:10.1038/s41467-021-21859-0)
Supplement: Supplementary file 10 — Source Data [file 41467_2021_21859_MOESM10_ESM.zip › Raw_data/readme.docx]

| Figure | Source data file |
| --- | --- |
| Figure 1g,h | RNA_seq_cpm_stats_raw |
| Figure2b | RNA_seq_cpm_stats_raw |
| Figure 3i | Figure_3i_raw |
| Figure 3j | Figure_3j_raw |
| Figure 4b | Figure_4_raw |
| Figure 5a | Figure_5_raw |
| Figure 6a | RNA_seq_cpm_stats_raw |
| Figure 6,b,c,d,e,g | Figure_6_raw |
| Figure 7a | Figure7a_raw |
| Supplementary Figure 1a | SF1_raw |
| Supplementary Figure 4a,b | RNA_seq_cpm_stats_raw |
| Supplementary Figure 8a,b,c,d | SF8_raw |
| Supplementary Figure 9c | SF9_raw |
| Supplementary Figure 10 | RNA_seq_cpm_stats_raw |

**List of figure that have associated source data.**
